# The Leukocyte Landscape Across Mouse Strains and Tissues

**DOI:** 10.1111/imcb.70168

**Published:** 2026-10-08

**Authors:** Ella Trolio, Sarah Dart, Liu Liu, Michaela Lucas, Amy Prosser

**Affiliations:** ^1^ Medical School, University of Western Australia Perth Australia; ^2^ Department of Immunology Sir Charles Gairdner Hospital, PathWest Perth Australia

**Keywords:** flow cytometry immunophenotyping, leukocyte composition, mouse strain differences, murine immune system, sex differences in immunity, tissue‐specific immunity

## Abstract

With widespread use of mice ( *Mus musculus* ) in immunological research, understanding baseline immune variation between mouse models is critical. While peripheral blood immune profiles have been well‐defined, strain‐ and sex‐specific differences in leukocyte composition across other tissues remain poorly characterized. Here, we performed flow cytometric immunophenotyping of 19 leukocyte subsets and 3 T cell memory phenotypes in the blood, bone marrow, liver, spleen, and lymph nodes of seven strains of naïve adult male mice: Balb.b, BALB/c, BALB/c Ly5.1, C57BL/6J, C57BL/6J Ly5.1, BALB/c:B6J (F1), and DBA/2 J. Parallel analyses were conducted in female BALB/c and C57BL/6J mice. Each tissue contained a unique immunobiome, with conventional T and B cells enriched in the lymph nodes and spleen, unconventional T cells and innate lymphoid cells (ILCs) in the liver, and myeloid cells in the bone marrow. Generally, BALB‐derived strains displayed a helper T cell‐ and innate‐skewed profile, enriched for CD4 and regulatory T cells, neutrophils, and natural killer cells. C57BL/6J‐derived strains were typically enriched for CD8 T cells, ILC1s, and conventional dendritic cells, consistent with a cytotoxic immune bias. Small differences were detected between congenic and parental strains. F1's exhibited a mix of C57BL/6J‐biased and intermediate parental phenotypes. DBA/2 J mice were immunologically distinct, notably enriched for γδ T cells. Sex‐associated differences were minor compared to strain effects. These findings provide tissue‐specific benchmarks for leukocyte composition across commonly used mouse strains. They also highlight the importance of strain and sex selection and baseline immunophenotyping to support accurate interpretation of observed immune phenotypes in experimental contexts.

## Graphical abstract

The blood, bone marrow, liver, lymph nodes, and spleen of naïve male mice contained unique immunobiomes which differed between commonly used strains. BALB‐derived strains exhibited helper‐ and innate‐biased immune profiles, C57BL/6J‐derived strains were enriched for cytotoxic and immune surveillance populations, and DBA/2 J mice displayed elevated γδ T cells. Sex‐associated differences were observed but were minor compared to strain differences. These findings establish a baseline reference for tissue‐specific immune variation and highlight the importance of mouse strain selection in immunological studies.

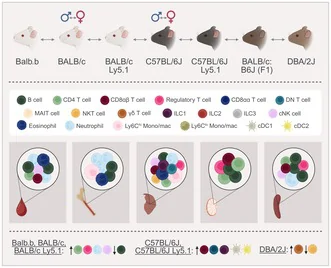

## 1 Introduction

Mouse ( *Mus musculus* ) models are a widely used resource in immunological research. These diverse models have enabled a broader understanding of immune regulatory mechanisms and have contributed substantially to advancing treatment of immune‐mediated diseases, including cancer, infections, and organ transplant rejection [1]. However, there are inherent immunological differences between commonly used mouse strains that can confound data interpretation and limit the translation of experimental findings if not carefully considered.

Immune‐related differences between inbred mouse strains arise from genetic variation accumulated through generations of selective breeding [2, 3]. These genetic differences result in distinct leukocyte compositions and signaling pathway biases, leading to strain‐dependent variation in disease susceptibility, progression, and outcome [4, 5]. In addition, male and female mice of the same strain can exhibit divergent immune responses, driven by both sex chromosome genetics and sex hormones, which differentially regulate immune cell function [6].

The leukocyte composition of murine peripheral blood has previously been comprehensively assessed and compared in over 30 mouse strains and between sexes, providing strain‐ and sex‐specific benchmarks for leukocyte population frequencies under normal physiological conditions [7, 8, 9, 10, 11]. Significant differences between strains have been highlighted; for example, PL/J and SJL/J mice have up to 20‐ to 30‐fold higher frequencies of CD4 and CD8 T cells than C57BL/10 J and NON/LtJ mice [7]. However, there is limited characterization of leukocyte populations in tissues other than blood [12]. Such information is timely given the increasing focus on tissue‐resident lymphocyte populations [13, 14, 15, 16], and is essential for accurately identifying altered immune phenotypes in disease models or following experimental interventions, and to make unbiased comparisons between studies using different mouse strains or sexes.

Here, we have examined 19 leukocyte subsets in the blood, liver, bone marrow (BM), spleen, and lymph nodes (LN) of adult males of seven mouse strains: Balb.b, BALB/c, BALB/c Ly5.1, C57BL/6J, C57BL/6J Ly5.1, BALB/c:B6J F1 hybrids, and DBA/2 J. These tissues and strains are broadly used in immunological research, but are of particular interest in solid organ transplantation studies. BALB/c (H‐2^d^) and C57BL/6J (H‐2^b^) mice represent a fully major histocompatibility complex (MHC) mismatched donor and recipient pairing widely used to model acute allograft rejection [17]. Balb.b mice share the genetic background of BALB/c mice but express the C57BL/6 MHC haplotype, providing a model to investigate MHC‐specific effects independent of broader genetic differences [17]. In contrast, BALB/c and DBA/2 J mice share the H‐2^d^ haplotype but differ at multiple minor histocompatibility loci, enabling analysis of minor antigen‐driven alloimmunity [18]. Ly5.1 congenic strains facilitate tracking of donor‐ and recipient‐derived leukocytes [19], while BALB/c × C57BL/6J F1 hybrids represent a semiallogeneic model frequently used in studies of immune tolerance and graft‐versus‐host disease [20]. Collectively, these strains encompass key strains and donor–recipient combinations commonly used in transplantation and other immunological research.

The leukocyte subsets evaluated include lymphoid and myeloid populations: T cells, both conventional (CD8αβ and CD4) T cells including regulatory T (Treg) cells, and unconventional T cells, including natural killer T (NKT) cells, mucosal‐associated invariant T (MAIT) cells, γδ T cells, CD8α^+^β^−^ (CD8αα) T cells, and TCRβ^+^CD4^−^CD8^−^ double‐negative (DN) T cells; innate lymphoid cells (ILCs), including conventional natural killer (cNK) cells, ILC1, ILC2, and ILC3 populations; B cells; Ly6C^hi^ and Ly6C^lo^ monocytes/macrophages; neutrophils; eosinophils; and type 1 and type 2 conventional dendritic cells (cDCs). Naïve (T_n_), central memory (T_cm_), and effector memory (T_em_) phenotypes were also assessed within CD4^+^ and CD8^+^ T cell populations. Further, these leukocyte subsets and phenotypes were compared between male and female BALB/c and C57BL/6J mice. The immune composition of all tissues varied markedly between strains, with comparatively minor effects of sex, revealing distinct tissue‐specific immune biases that are present in a naïve state. Overall, these findings emphasize the importance of strain and sex selection in mouse studies and provide a valuable point of reference and framework for immunological experimental design.

## 2 Methods

### 2.1 Animals

Male and female wildtype C57BL/6J and BALB/c, and male B6.SJL‐Ptprc^a^Pepc^b^/BoyJ (referred to as C57BL/6J Ly5.1), BALB/c:B6J (CB6F1, referred to as F1), Balb.b (CB10‐H2b/LilMcdJ), and DBA/2 J (DBA/2 Jarc) mice were purchased from the Animal Resources Centre (ARC, later Ozgene‐ARC, Perth, Western Australia). CByJ.SJL (B6)‐Ptprc^a^/J (referred to as BALB/c Ly5.1) mice were imported from The Jackson Laboratory (stock #006584) and bred at ARC/Ozgene‐ARC. BALB/c Ly5.1 mice used in this study were derived from the 3rd to 6th generation of in‐house breeding, with the colony housed for ~9–30 months prior to tissue collection. All mice were housed under specific pathogen free conditions at a maximum of five per cage, fed normal chow and had access to water ad libitum. Mice were between 13 and 22 weeks of age, with intra‐ and inter‐strain variation due to their inclusion in concurrent experiments in which mice were weight‐matched to those undergoing heart (~20 g) or liver (~25–30 g) transplantation. All mice were drug and test naïve, and had at least 7 days of acclimatization prior to tissue collection.

### 2.2 Sample Collection and Tissue Processing

The blood, hindlimb BM, liver, spleen, and abdominal LN (mesenteric, pyloric, pancreatic, renal, lumbar, and caudal) were collected from each mouse. All tissues were processed using mechanical dissociation to isolate leukocytes while preserving surface marker integrity, as previously described [21, 22]. The liver was perfused with 10 mL of cold saline prior to resection to remove circulating leukocytes. All tissues were passed through 70 μm cell strainers to form single cell suspensions. Leukocytes were enriched from the liver suspension by 42% Percoll (Cytiva Life Sciences, Marlborough, MA) density‐gradient centrifugation. Red cells were lysed from all samples using 1× Red Blood Cell Lysis Buffer (Tonbo Biosciences, San Diego, CA) as per the manufacturer's instructions. Leukocytes were counted using Countess II FL or Countess 3 FL Automated Cell Counters (Thermo Fisher, Waltham, MA).

### 2.3 Flow Cytometry Analysis

Viability was determined by staining with Zombie UV Fixable Viability Dye (BioLegend, 1:1000, San Diego, CA). Leukocytes (0.1–4 × 10^6^) were stained with antibodies for three flow cytometry panels, listed in Table 1. Cells used for myeloid panel staining were first incubated with anti‐CD16/32 (Becton Dickinson: BD, Franklin Lakes, NK, 1:50) for 5 min at 4°C. Intracellular antibody staining was performed using the Foxp3/Transcription Factor Staining Buffer Set (Thermo Fisher), as per the manufacturer's instructions. After staining, cells were fixed with Stabilizing Fixative (BD), and data were acquired on a BDSORP LSRFortessa flow cytometer (BD). Prior to each acquisition, quality control was performed using Cytometer Setup and Tracking beads (BD) and Ultra Rainbow Calibration Particles (Spherotech, Lake Forest, IL), and application settings were used. Data were analyzed using Flowjo software (BD), first subject to FlowAI [23] to remove anomalies. Leukocyte subset gating strategies are shown in Figures S1–S3.

**TABLE 1:** Flow cytometry antibody details.

Antibody (clone)	Source	Identifier	Dilution
Panel 1: B and T lymphocytes			
CD19 Brilliant Violet 480 (1D3)	BD Biosciences	AB_2739509	1:25
CD1d tetramer APC (αGalCer loaded)	NIH	NA	1:200
CD4 Brilliant Violet 786 (GK1.5)	BD Biosciences	AB_2738140	1:1000
CD44 Brilliant Blue 515 (IM7)	BD Biosciences	AB_2738855	1:100
CD45.1 Brilliant Violet 711 (A20)	BD Biosciences	AB_2861197	1:200
CD45.2 APC/eFluor 780 (104)	Thermo Fisher	AB_1272175	1:400
CD49a Brilliant Blue 700 (Ha31/8)	BD Biosciences	AB_2861198	1:100
CD62L eVolve 605 (MEL‐14)	Thermo Fisher	AB_2574718 (discontinued)	1:4000
CD62L Super Bright 600 (MEL‐14)	Thermo Fisher	AB_2637416	1:4000
CD69 PE/Cy7 (H1.2F3)	BD Biosciences	AB_394508	1:40
CD8α Super Bright 645 (53–6.7)	Thermo Fisher	AB_2662353	1:200
CD8β Brilliant UV 395 (H35‐17.2)	BD Biosciences	AB_2740017	1:2000
FoxP3 PE/CF594 (MF23)	BD Biosciences	AB_11151905	1:50
MR1 tetramer Brilliant Violet 421 (5‐OP‐RU)	NIH	NA	1:200
TCRβ AlexaFluor 700 (H57‐597)	BioLegend	AB_1027648	1:50
TCRγδ PE (GL3)	BD Biosciences	AB_394689	1:100
Panel 2: Innate lymphoid cells			
CD117 (c‐kit) Brilliant Violet 786 (2B8)	BD Biosciences	AB_2732005	1:100
CD11c AlexaFluor 700 (N418)	Thermo Fisher	AB_493993	1:200
CD127 PE/CF594 (SB1199)	BD Biosciences	AB_11153131	1:20
CD200R AlexaFluor 647 (OX110)	BD Biosciences	AB_2739701	1:100
CD45.1 APC/eFluor 780 (A20)	Thermo Fisher	AB_1582228	1:200
CD45.2 FITC (104)	BD Biosciences	AB_395041	1:100
CD49a Brilliant Violet 510 (H93118)	BD Biosciences	AB_2739900	1:50
CD49b PE (DX5)	BD Biosciences	AB_395094	1:1000
Eomes PE/Cy7 (Dan11mag)	Thermo Fisher	AB_2573453	1:100
GATA‐3 Brilliant Violet 421 (L50‐823)	BD Biosciences	AB_2738152	1:20
Mouse lineage cocktail (Lin) PerCP/Cy5.5	BD Biosciences	AB_10612020	1:10
NK1.1 Brilliant UV 395 (PK136)	BD Biosciences	AB_2738618	1:200
NKp46 Brilliant UV 395 (29A1.4)	BD Biosciences	AB_2740061	1:20
RORγt Brilliant Violet 650 (Q31‐378)	BD Biosciences	AB_2738915	1:50
T‐bet Brilliant Violet 605 (4B10)	BioLegend	AB_11219388	1:20
TCRγδ Brilliant Violet 711 (GL3)	BD Biosciences	AB_2738531	1:50
Panel 3: Myeloid cells			
B220 AlexaFluor 700 (RA3‐6B2)	Thermo Fisher	AB_891458	1:50
CD11b eVolve 605 (M1/70)	Thermo Fisher	AB_2574700 (discontinued)	1:4000
CD11b Super Bright 600 (M1/70)	Thermo Fisher	AB_2637408	1:4000
CD11c PE/CF594 (HL3)	BD Biosciences	AB_2737617	1:100
CD206 Brilliant Violet 711 (C068C2)	BioLegend	AB_2565822	1:50
CD3 PerCP/eFluor 710 (17A2)	Thermo Fisher	AB_1834427	1:200
CD45.1 PE (A20)	BD Biosciences	AB_10897174	1:500
CD45.2 APC/eFluor 780 (104)	Thermo Fisher	AB_1272175	1:200
F4/80 AlexaFluor 647 (T45‐2342)	BD Biosciences	AB_2744474	1:100
Ki67 PE/Cy7 (B56)	BD Biosciences	AB_10716060	1:100
Ly6C Brilliant Violet 510 (HK1.4)	BioLegend	AB_2562351	1:1000
Ly6G FITC (1A8)	BD Biosciences	AB_394207	1:100
MHC Class II (I‐A/I‐E) eVolve 655 (M5/114.15.2)	Thermo Fisher	AB_2574798	1:500
MR1 tetramer Brilliant Violet 421 (5‐OP‐RU)	NIH	NA	1:400
NKp46 AlexaFluor 700 (29A1.4)	BD Biosciences	AB_10561840	1:50
Siglec‐F Brilliant Violet 786 (E50‐2440)	BD Biosciences	AB_2740581	1:200

### 2.4 Quantification and Statistical Analysis

Statistical analyses were performed in Prism, v10 (GraphPad Software, San Diago, CA). Data were first tested for outliers (robust regression followed by outlier identification analysis), homogeneity of variance (Levene's or Bartlett's test), and normal distribution (Shapiro–Wilk test). Significant differences in individual leukocyte populations between strains were identified using Ordinary One‐Way or Welch's ANOVA, or Kruskal–Wallis tests, followed by Tukey's, Dunnett's, or Dunn's multiple comparisons corrections. Significant differences in individual leukocyte populations between sexes were identified using unpaired or Welch's *t*‐tests, or Mann–Whitney tests, using the two‐stage Benjamini, Krieger, and Yekutieli procedure to control the false discovery rate. Data are presented as mean + SEM.

## 3 Results

### 3.1 BALB‐Derived Strains Have Reduced B Cell Frequencies and a Helper T Cell Bias Compared to CD8αβ‐Enriched C57BL/6J‐Derived and F1 Mice

To characterize tissue‐specific immune populations across mouse strains, we immunophenotyped 13 lymphoid and 6 myeloid populations in the blood, BM, liver, LN, and spleen of naïve, adult male mice. All populations were quantified as proportions of CD45^+^ leukocytes to enable cross‐tissue comparisons. Subset numbers were also calculated from total cell counts and are provided in Tables S1–S5.

We first focused on B cells and conventional TCRβ^+^ T cells (Figure 1). As expected, these subsets were enriched in secondary lymphoid organs, with the frequency of B and T cells greatest in the LN, then spleen, blood, liver, and lowest in the BM. B cells were generally most abundant in the spleen, with lower but similar frequencies in the other tissues. CD8αβ and CD4 T cells were enriched in the LN, with lower frequencies found in the spleen, liver, and blood, and few found in the BM. Tregs were present in all tissues at low frequencies, though they were most abundant in the LN in all mouse strains.

**FIGURE 1:**
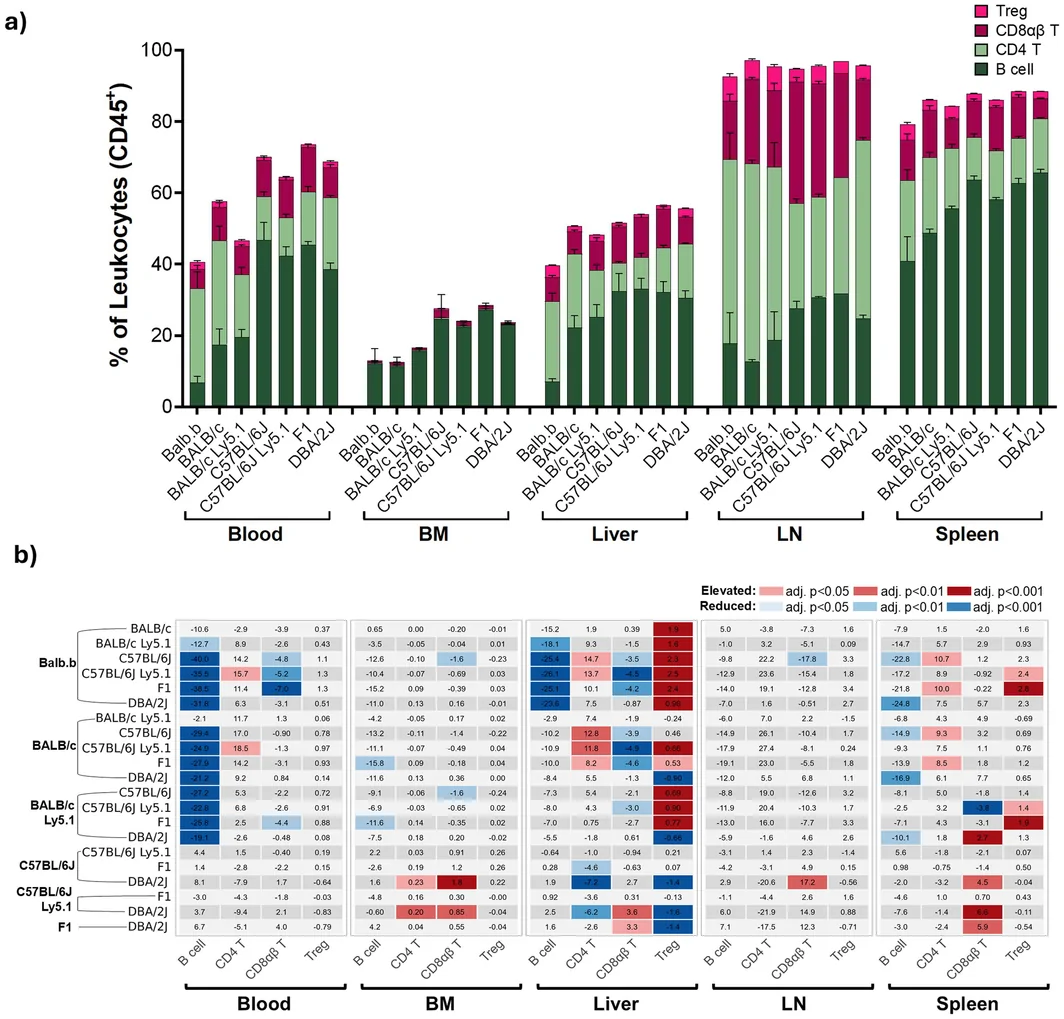
B and CD8αβ T cells are lower, but CD4 T cells higher in BALB‐derived mice, compared to C57BL/6‐derived and F1 mice. (a) Frequencies of conventional T and B cell subsets in the blood, bone marrow (BM), liver, lymph nodes (LN), and spleen. (b) Heatmap of pairwise differences between strains. Values represent magnitude of change in frequency. Positive values indicate enrichment and negative values indicate reduction in the bracket title strain relative to the bracketed strain. Flow cytometry data were acquired across multiple days with pre‐acquisition quality controls to ensure consistency across runs. Significant differences were identified using ordinary one‐way or Welch's ANOVA, or Kruskal–Wallis tests, followed by Tukey's, Dunnett's, or Dunn's multiple comparisons corrections. Data are presented as mean + SEM. Balb.b, *n* = 5; BALB/c, *n* = 4; BALB/c Ly5.1, *n* = 6 (LN, *n* = 3); C57BL/6J, *n* = 5 (LN, *n* = 4); C57BL/6J Ly5.1, *n* = 8 (LN, *n* = 3); F1 (BALB/c:B6J), *n* = 5 (LN, *n* = 1); and DBA/2 J, *n* = 5. Unshaded cells indicate nonsignificant comparisons (adj. *p* > 0.05). Treg, regulatory T cell.

Strain‐specific differences were most pronounced in the liver, then spleen, blood, and BM, while the composition of conventional T and B cells in the LN was remarkably consistent across strains. For B cells, pronounced strain‐dependent variation in frequency was observed, primarily in the blood, liver, and spleen (Figure 1b). Generally, lower B cell frequencies were observed in BALB‐derived strains (Balb.b, BALB/c, and BALB/c Ly5.1), compared with C57BL/6J‐derived (C57BL/6J and C57BL/6J Ly5.1), F1, and DBA/2 J mice (Figure 1a). For example, B cells comprised 7% ± 2% of CD45^+^ leukocytes in the blood of Balb.b mice compared to 47% ± 5% in C57BL/6J mice, and 11% ± 2% in the BM of BALB/c mice compared to 27% ± 2% in F1 mice.

Total TCRβ^+^ T cell frequencies were broadly comparable between strains within each tissue; however, some differences were observed, particularly in the liver and blood (Figure S4). More substantial differences were observed between CD4 T, CD8αβ T, and Treg subsets (Figure 1b). For CD4 T cells, inter‐strain differences were most pronounced in the liver and spleen, with the blood, BM, and LN more consistent across strains (Figure 1b). C57BL/6J‐derived and F1 mice generally displayed reduced CD4 T cell frequencies relative to BALB‐derived strains and DBA/2 J mice, particularly Balb.b and BALB/c mice (Figure 1a). For example, CD4 T cells 8% ± 1% of leukocytes in the liver of C57BL/6J mice compared to comprised 21% ± 1% in BALB/c mice, and 11% ± 1% in the blood of C57BL/6J Ly5.1 mice compared to 26% ± 5% in Balb.b mice.

The distribution of CD8αβ T cells also differed between strains, being most pronounced in the liver and spleen, with few differences detected in the blood, BM and LN (Figure 1b). C57BL/6J‐derived and F1 mice generally exhibited higher CD8αβ T cell frequencies than BALB‐derived strains and DBA/2 J mice (Figure 1a). For example, CD8αβ T cells constituted 34% ± 1% of leukocytes in the LN of C57BL/6J mice compared with 17% ± 0.5% in DBA/2 J mice, and 12% ± 1% in the blood of F1 mice versus 8% ± 1% in BALB/c Ly5.1 mice.

Tregs represented a minor fraction of total T cells across strains and tissues, for example, ranging from 2% to 8% in the liver and 6% to 11% in the spleen (Figure 1a). A similar trend to CD4 T helper cells was observed, where BALB‐derived strains, particularly Balb.b mice, had greater Treg frequencies compared to C57BL/6J‐derived and F1 mice. Significant differences between strains were largely restricted to the relatively small Treg populations in the liver (Figure 1b).

### 3.2 DBA/2 J, C57BL/6J‐Derived, and BALB‐Derived Mice Have Divergent Unconventional T Cell Biases

We next assessed unconventional T cell populations (Figure 2). All five subsets (CD8αα, DN, MAIT, NKT, and γδ T cells) were enriched in the liver, particularly NKT, γδ T, and DN T cells, which comprised up to 23%, 13%, and 8% of total hepatic T cells, respectively (8%, 4%, and 3.5% of leukocytes). Smaller but robust populations of unconventional T cells were detected in all other tissues, where they comprised < 3% of leukocytes. While many strain‐specific differences were observed, these were mostly due to relatively minor differences in low abundance subsets.

**FIGURE 2:**
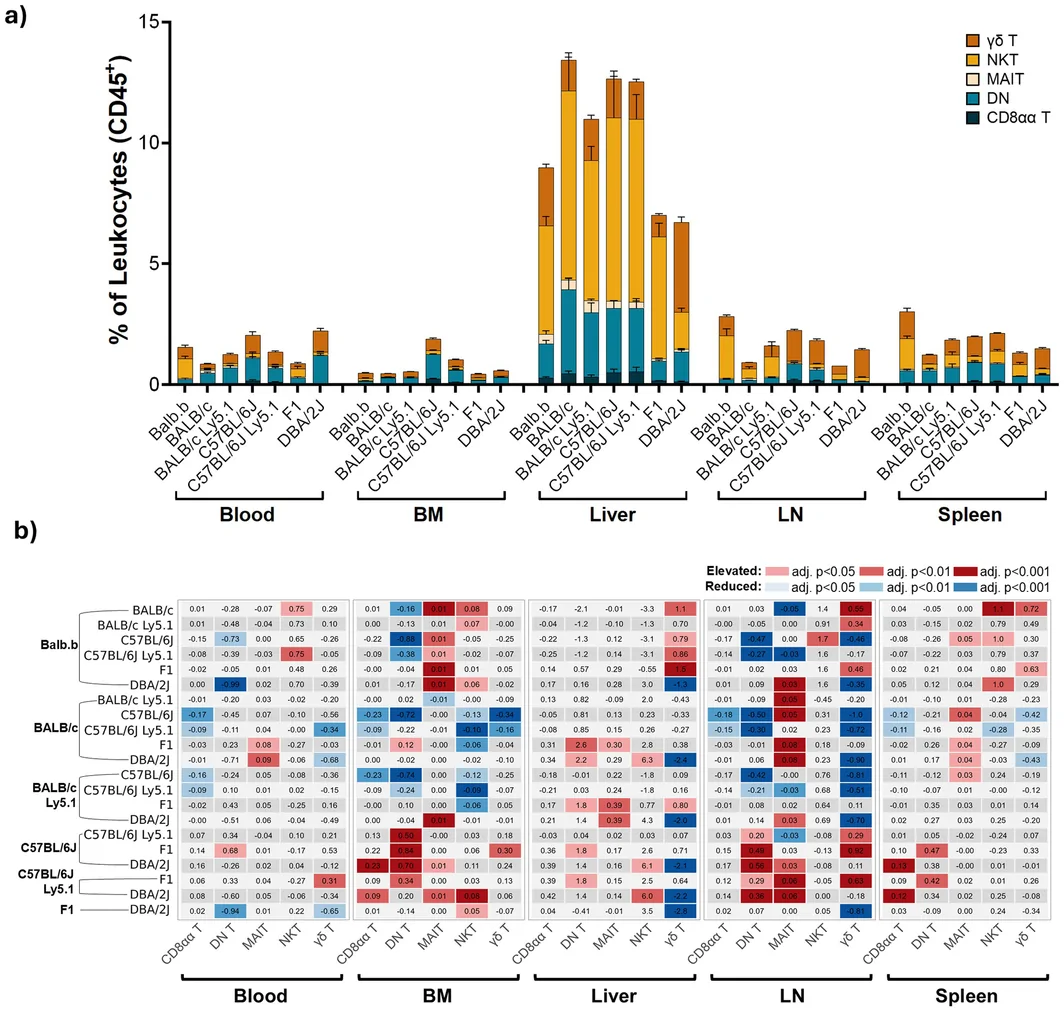
Unconventional T cell subsets are differentially enriched across mice from DBA/2 J, C57BL/6J, and BALB backgrounds. (a) Proportion of unconventional T cell subsets in the blood, bone marrow (BM), liver, lymph nodes (LN), and spleen. (b) Heatmap of pairwise differences between strains. Values represent magnitude of change in frequency. Positive values indicate enrichment and negative values indicate reduction in the bracket title strain relative to the bracketed strain. Flow cytometry data were acquired across multiple days with pre‐acquisition quality controls to ensure consistency across runs. Significant differences were identified using ordinary one‐way or Welch's ANOVA, or Kruskal–Wallis tests, followed by Tukey's, Dunnett's, or Dunn's multiple comparisons corrections. Data are presented as mean + SEM. Balb.b, *n* = 5; BALB/c, *n* = 4; BALB/c Ly5.1, *n* = 6 (LN, *n* = 3); C57BL/6J, *n* = 5 (LN, *n* = 4); C57BL/6J Ly5.1, *n* = 8 (LN, *n* = 3); F1 (BALB/c:B6J), *n* = 5 (LN, *n* = 1); and DBA/2 J, *n* = 5. Unshaded cells indicate nonsignificant comparisons (adj. *p* > 0.05). NKT, natural killer T cell; MAIT, mucosal‐associated invariant T cell; DN, TCRβ^+^CD4^−^CD8^−^ double negative T cell.

γδ T cells were one of the larger unconventional T cell populations detected across all tissues (Figure 2a). They exhibited marked strain‐dependent differences, particularly in the LN and liver (Figure 2b). Notably, γδ T cells were significantly more enriched in the livers of DBA/2 J mice compared to all other strains (3.7% ± 0.2% vs. 0.9% ± 0.1% to 2.4% ± 0.2%), with enrichment in DBA/2 J also seen in the blood and LN.

NKT cells were universally enriched in the liver (Figure 2a). Although not statistically significant, there was a trend toward reduced NKT cell frequencies observed in DBA/2 J mice across most tissues when compared to other mouse strains. While statistical inter‐strain differences in NKT proportions were found most in the BM, this was likely due to their low abundance, with fewer differences detected in other tissues (Figure 2b). Balb.b mice consistently had higher NKT cell frequencies in the blood, LN, and spleen when compared to all other strains. For example, NKT cells comprised 1.8% ± 0.3% of leukocytes in the LN and 1.3% ± 0.1% in the spleen of Balb.b mice, compared to 0.1% ± 0.01% and 0.3% ± 0.01% in C57BL/6J mice, respectively.

DN T cells were detected in every tissue and exhibited strain‐dependent differences in frequency, particularly in the LN and BM (Figure 2b). In these tissues, their abundance was generally higher in C57BL/6J‐derived strains compared with mice from other backgrounds (Figure 2a). In the BM, DN T cells accounted for 1% ± 0.1% of leukocytes in C57BL/6J mice, compared with 0.1% ± 0.08% in Balb.b mice. In the LN, frequencies were 0.7% ± 0.1% in C57BL/6J mice versus 0.2% ± 0.02% in BALB/c mice.

CD8αα T cells were a rare population across tissues (< 5% of T cells and < 0.6% of leukocytes), with few differences found in the blood, BM, LN, and spleen, and no significant differences in the liver (Figure 2b). Like CD8αβ T cells, CD8αα T cell frequencies were generally greater in C57BL/6J‐derived strains compared to other strains (Figure 2a).

MAIT cells constituted the smallest fraction of T cells across tissues. While statistically significant differences in the frequency of MAIT cells were detected (Figure 2b), these were small in magnitude and likely reflect the low abundance of this subset.

### 3.3 Mouse Strain Influences naïve and Memory CD4 and CD8 T Cell Composition

To further characterize T cell phenotype, we assessed the proportions of T_n_ (CD44^−^CD62L^+^), T_cm_ (CD44^+^CD62L^+^), and T_em_ (CD44^+^CD62L^−^) subsets within the CD69^−^CD4^+^ and CD69^−^CD8^+^ T cell populations (Figure 3; Figure S5). Consistent with their function as secondary lymphoid organs, the LN was composed predominantly of naïve CD4 (77%–91%) and CD8 (73%–91%) T cells, with only minor T_cm_ and T_em_ populations. The spleen and blood also contained predominantly naïve T cells but exhibited modestly larger memory compartments than the LN. In contrast, the BM and liver had reduced frequencies of naïve T cells and increased proportions of memory subsets. For CD4 T cells, both T_cm_ (9%–34%) and more prominently, T_em_ (24%–60%) cells were enriched in the BM, while the liver also contained high proportions of CD4 T_em_ cells (13%–27%). For CD8 T cells, T_cm_ cells were enriched in the BM (5%–43%), whereas T_em_ cells were most abundant in the liver (8%–17%).

**FIGURE 3:**
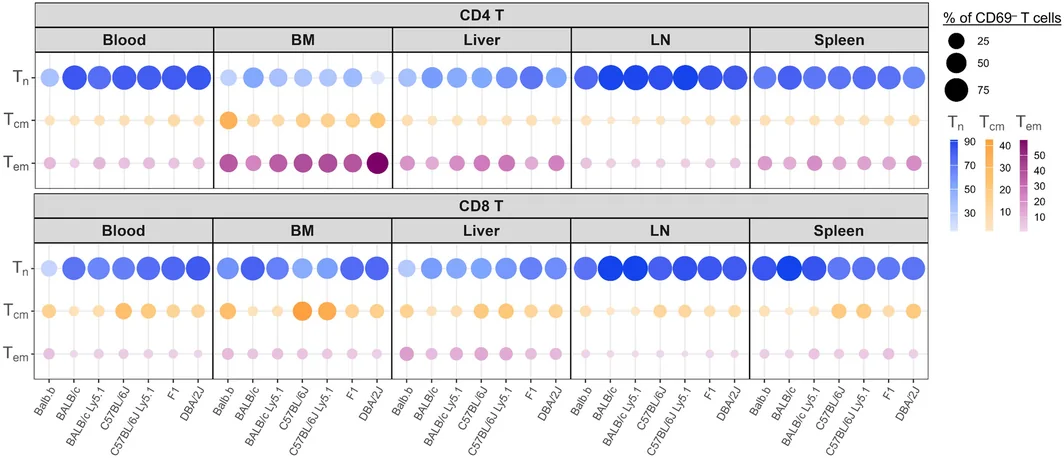
BALB‐, C57BL/6J‐, and DBA‐derived mouse strains exhibit distinct naïve and memory T cell phenotypes. Percentage (%) of naïve (T_n_), central memory (T_cm_), and effector memory (T_em_) T cell subsets of CD69^−^ CD4 and CD8 T cells in the blood, bone marrow (BM), liver, lymph nodes (LN), and spleen. Flow cytometry data were acquired across multiple days with pre‐acquisition quality controls to ensure consistency across runs. Data are presented as mean frequency. Balb.b, *n* = 5; BALB/c, *n* = 4; BALB/c Ly5.1, *n* = 6 (LN, *n* = 3); C57BL/6J, *n* = 5 (LN, *n* = 4); C57BL/6J Ly5.1, *n* = 8 (LN, *n* = 3); F1 (BALB/c:B6J), *n* = 5 (LN, *n* = 1); and DBA/2 J, *n* = 5.

For CD4 T cells, strain‐specific differences in memory phenotypes were most evident in the spleen and liver, whereas variation was greatest in the spleen, blood, and BM for CD8 T cells (Figure S5). In general, CD4 T_n_ frequencies were lowest in Balb.b mice and were consistently reduced compared with BALB/c mice (Figure 3). For example, T_n_ comprised 37% ± 6% of CD69^−^ CD4 T cells in the blood of Balb.b mice compared to 84% ± 1% in BALB/c mice. Similarly, CD8 T_n_ frequencies were generally reduced in Balb.b mice in the blood, liver, and LN. In contrast, in the BM and spleen, the lowest CD8 T_n_ frequencies were observed in C57BL/6J and C57BL/6J Ly5.1 mice. For example, T_n_ cells comprised 68% ± 3% of CD69^−^ CD8 T cells in the BM and 85% ± 1% in the spleen of BALB/c Ly5.1, compared to 49% ± 2% and 71% ± 2% in C57BL/6J mice, respectively.

For CD4 T_cm_ cells, higher frequencies were observed in DBA2/J mice within the LN and spleen and in Balb.b mice within the BM and liver, particularly compared to BALB/c mice (Figure 3; Figure S5). For example, T_cm_ cells comprised 5% ± 0.8% of CD69^−^ CD4 T cells in the LN of DBA/2 J mice compared with 1% ± 0.1% in BALB/c mice. In contrast, CD8 T_cm_ cells were enriched in C57BL/6J and C57BL/6J Ly5.1 mice compared with BALB/c and BALB/c Ly5.1 mice across tissues, with F1 and DBA/2 J strains generally exhibiting intermediate frequencies. This was most pronounced in the BM with frequencies of T_cm_ cells reaching 43% ± 3% in C57BL/6J mice compared with 5% ± 0.6% in BALB/c mice.

No consistent strain‐specific pattern was evident for CD4 T_em_ cells, with frequencies varying between strains in a tissue‐specific manner (Figure 3; Figure S5). For CD8 T_em_ cells, there was a trend toward higher frequencies in Balb.b mice across all tissues except the spleen, although this difference was only statistically significant in the blood, where frequencies reached 7% ± 1% compared with 1% ± 0.1% in BALB/c and 2% ± 0.1% in DBA2/J mice.

### 3.4 ILC1s Are Enriched in C57BL/6J‐Derived Mice While cNK Cells Are Enriched in Balb.b and BALB/c Mice

We next examined ILC composition across tissues (Figure 4). NK cells were identified using both NK1.1 and NKp46 staining, as BALB/c‐derived strains lack NK1.1 expression. Across C57BL/6 Ly5.1 mice, a strain in which the Ncr1/NKp46 mutation has previously been reported in some colonies, distinct NK1.1‐ and NKp46‐positive populations were observed (Figure S2), indicating that the mice assessed were not affected. Like unconventional T cells, ILCs were enriched in the liver, though ILC2 and ILC3 populations were infrequent across all tissues examined. cNK cells were the dominant ILC subset across tissues, which after the liver were most frequent in the spleen then blood, with low frequencies in the BM and LN. ILC1s were largely restricted to the liver, where they constituted up to 9% of leukocytes, and were rare in other tissues.

**FIGURE 4:**
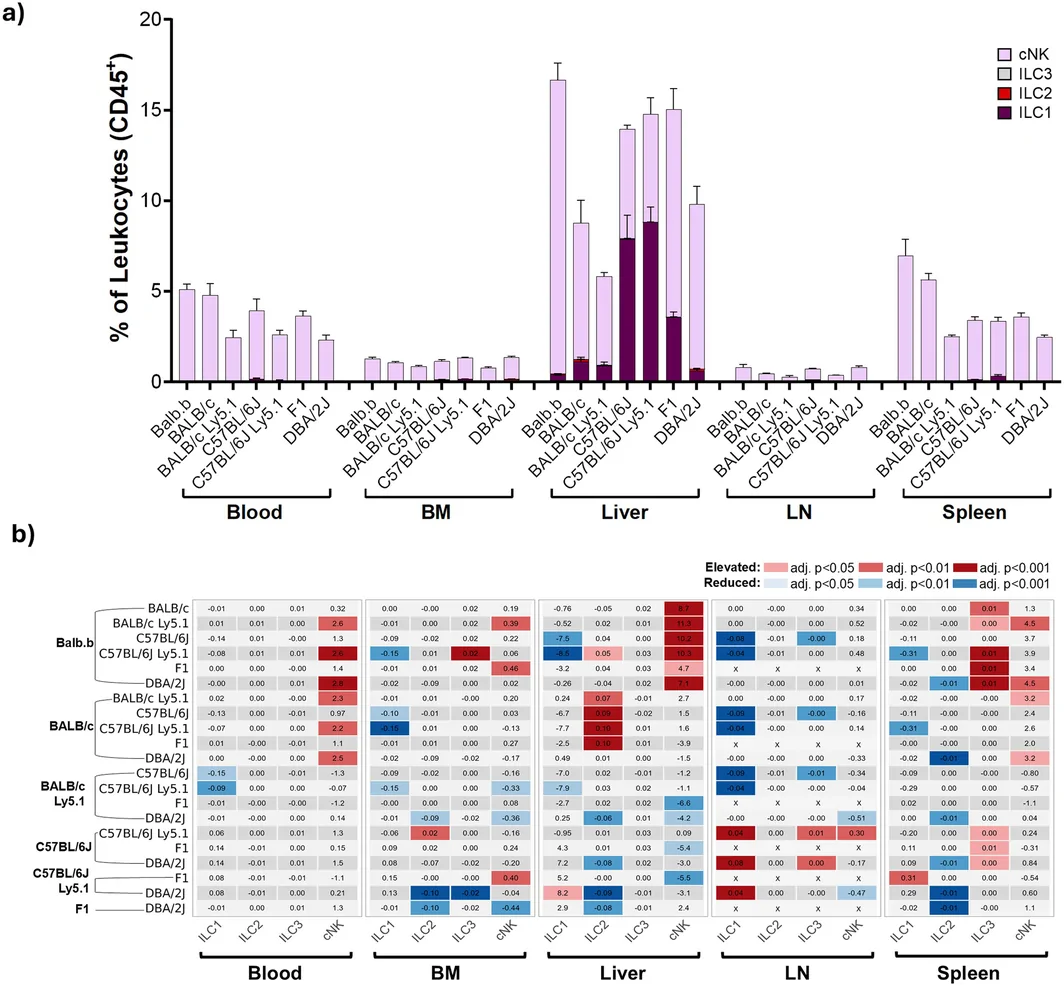
Type 1 innate lymphoid cells (ILCs) are enriched in C57BL/6J‐derived mice while conventional natural killer cells (cNKs) are enriched in Balb.b and BALB/c mice. (a) Proportion of ILC subsets in the blood, bone marrow (BM), liver, lymph nodes (LN), and spleen. (b) Heatmap of pairwise differences between strains. Values represent magnitude of change in frequency. Positive values indicate enrichment and negative values indicate reduction in the bracket title strain relative to the bracketed strain. Flow cytometry data were acquired across multiple days with pre‐acquisition quality controls to ensure consistency across runs. Significant differences were identified using ordinary one‐way or Welch's ANOVA, or Kruskal–Wallis tests, followed by Tukey's, Dunnett's, or Dunn's multiple comparisons corrections. Data are presented as mean + SEM. Balb.b, *n* = 5; BALB/c, *n* = 4; BALB/c Ly5.1, *n* = 6 (LN, *n* = 3); C57BL/6J, *n* = 5 (LN, *n* = 4); C57BL/6J Ly5.1, *n* = 8 (LN, *n* = 3); F1 (BALB/c:B6J), *n* = 3; and DBA/2 J, *n* = 5. Unshaded cells indicate non‐significant comparisons (adj. *p* > 0.05). X: Comparison not performed due to insufficient sample size.

Strain variation was most pronounced in the liver, followed by the spleen, LN, and BM, with minimal differences observed in the blood (Figure 4b). For cNK cells, marked inter‐strain differences were detected most prominently in the liver, then the blood and BM. In the blood, liver and spleen, cNK cells were typically enriched in Balb.b and BALB/c, but not BALB/c Ly5.1 mice, relative to C57BL/6J‐derived strains (Figure 4a). This was most pronounced in the liver, where cNK cells comprised 16% ± 1% of leukocytes in Balb.b mice compared to 6% ± 0.2% in C57BL/6J mice.

In contrast, ILC1 frequencies were consistently higher in C57BL/6J‐derived strains compared to mice from other backgrounds (Figure 4a). ILC1s comprised 0.2%–9% of leukocytes across tissues in C57BL/6J mice and 0.1%–8% in C57BL/6J Ly5.1 mice, compared with 0.01%–1% in all other strains. Although statistically significant differences were detected for ILC2 and ILC3 populations (Figure 4b), these changes were generally small in magnitude, and the populations comprised fewer than 0.3% of leukocytes.

### 3.5 BALB‐Derived Mice Have Higher Neutrophil Frequencies, While C57BL/6J‐Derived Mice Are Enriched for Ly6C^lo^ Monocytes/Macrophages and cDCs

We then analyzed myeloid populations (Figure 5). As expected, myeloid cells were most abundant in the BM followed by the blood, then liver and spleen, and were scarce in the LN. Neutrophils were the dominant subset in the BM and blood, with lower frequencies detected in the other tissues. Eosinophils were detected at low but comparable frequencies in the BM, blood, and liver, and were rare in the spleen and LN. The frequency of Ly6C^hi^ monocyte/macrophage populations was highest in the BM and liver, and Ly6C^lo^ monocyte/macrophages and cDCs were present in all tissues at low frequencies, though they were generally most abundant in the liver.

**FIGURE 5:**
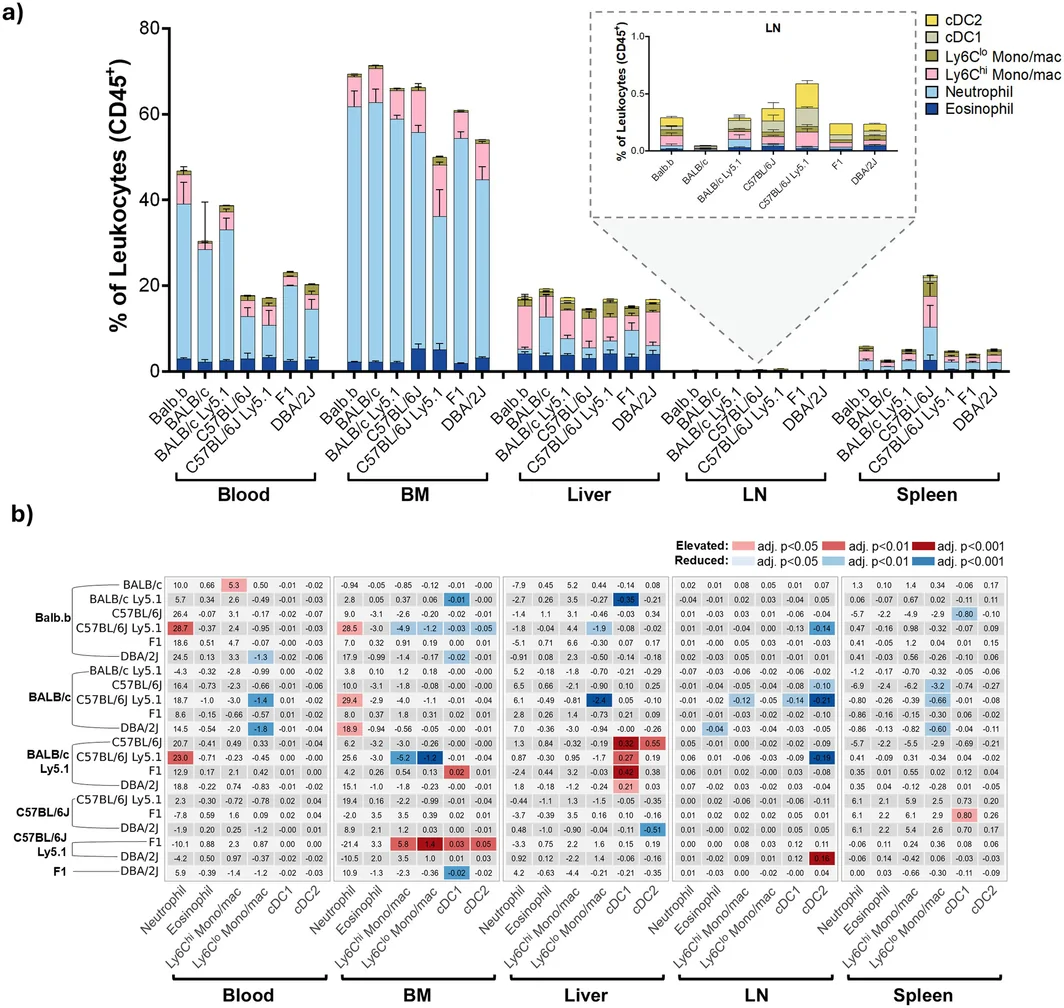
BALB‐derived mice have more neutrophils, while C57BL/6J‐derived mice are enriched for conventional dendritic cells (cDCs). (a) Proportion of myeloid cell subsets in the blood, bone marrow (BM), liver, lymph nodes (LN), and spleen. (b) Heatmap of pairwise differences between strains. Values represent magnitude of change in frequency. Positive values indicate enrichment and negative values indicate reduction in the bracket title strain relative to the bracketed strain. Flow cytometry data were acquired across multiple days with pre‐acquisition quality controls to ensure consistency across runs. Significant differences were identified using ordinary one‐way or Welch's ANOVA, or Kruskal–Wallis tests, followed by Tukey's, Dunnett's, or Dunn's multiple comparisons corrections. Data are presented as mean + SEM. Balb.b, *n* = 5; BALB/c, *n* = 4; BALB/c Ly5.1, *n* = 6 (LN, *n* = 3); C57BL/6J, *n* = 5 (LN and blood, *n* = 3); C57BL/6J Ly5.1, *n* = 8 (LN, *n* = 3); F1 (BALB/c:B6J), *n* = 3 (LN, *n* = 1); and DBA/2 J, *n* = 5 (spleen, *n* = 4). Unshaded cells indicate non‐significant comparisons (adj. *p* > 0.05). Mono/mac, monocyte/macrophage.

Overall, strain‐associated differences in myeloid composition were limited across tissues, and were most apparent in the BM and liver (Figure 5b). For neutrophils, significant differences in frequency between strains were only observed in the blood (7.5%–36% of leukocytes) and BM (31%–60%). In these tissues, BALB‐derived strains generally had higher neutrophil frequencies than C57BL/6J‐derived mice (Figure 5a). For example, neutrophils comprised 36% ± 5% of leukocytes in the blood of Balb.b mice compared with 8% ± 3% in C57BL/6J Ly5.1 mice. We also observed variation in the expression of the key surface proteins CD11b and Ly6G by neutrophil populations (Figure S6). Mouse neutrophils are typically defined as CD11b^+^Ly6G^+^, however, CD11b and Ly6G expression, measured by fluorescence intensity, varied between strains (Figure S7). For example, neutrophils from the blood and liver of Balb.b and F1 mice had minimal CD11b expression, while DBA/2 J neutrophils exhibited lower Ly6G expression than other strains across tissues.

Monocyte/macrophage populations (CD11b^+^F4/80^+^, Figure S3) were separated into Ly6C^hi^ and Ly6C^lo^ subsets. The frequency of Ly6C^hi^ populations differed minimally between strains, with significant differences observed in a tissue‐specific manner across the blood, BM, and LN (Figure 5b). There were also few significant differences in Ly6C^lo^ populations, which were observed primarily in the blood, BM, and spleen (Figure 5b). Generally, Ly6C^lo^ populations were more frequent in C57BL/6J Ly5.1 mice than in BALB‐derived strains, although their overall frequency remained < 3% across tissues (Figure 5a).

cDCs made up a relatively small part of the myeloid population in all tissues and the cDC2 population predominated over the cDC1 subset across tissues (Figure 5a). Strain‐associated differences in cDC1 frequencies were most prominent in the BM and liver, while variation in cDC2s was primarily detected in the BM and blood (Figure 5b). Both cDC subsets were generally enriched in C57BL/6J and/or C57BL/6J Ly5.1 mice compared with the other strains. For example, cDC1s comprised 0.9% ± 0.3% of leukocytes in the spleen of C57BL/6J mice compared with 0.1% ± 0.01% in F1 mice, while cDC2s accounted for 0.2% ± 0.03% in the LN of C57BL/6J Ly5.1 mice versus 0.01% ± 0.001% in BALB/c mice. This was observed across tissues except for the liver, where both subsets were enriched in BALB/c Ly5.1 mice.

### 3.6 Sex‐Associated Differences in Leukocyte Composition Are Modest Relative to Strain Effects

In addition to strain effects, we examined sex‐associated differences in immune composition between male and female C57BL/6J and BALB/c mice. Overall, sex‐related differences in leukocyte profiles were limited and largely tissue‐specific (Figure 6). In female BALB/c mice, LN contained fewer CD8αβ and CD4 T cells but higher frequencies of CD8αα T cells, γδ T cells and ILC1s compared to males. In the BM, females exhibited increased γδ T cells and Ly6C_lo_ monocytes/macrophages alongside reduced frequencies of ILC1 and MAIT cells. B cells were also enriched in the spleen of female BALB/c mice compared to male mice. No statistically significant differences were detected between tissues of male and female C57BL/6J mice.

**FIGURE 6:**
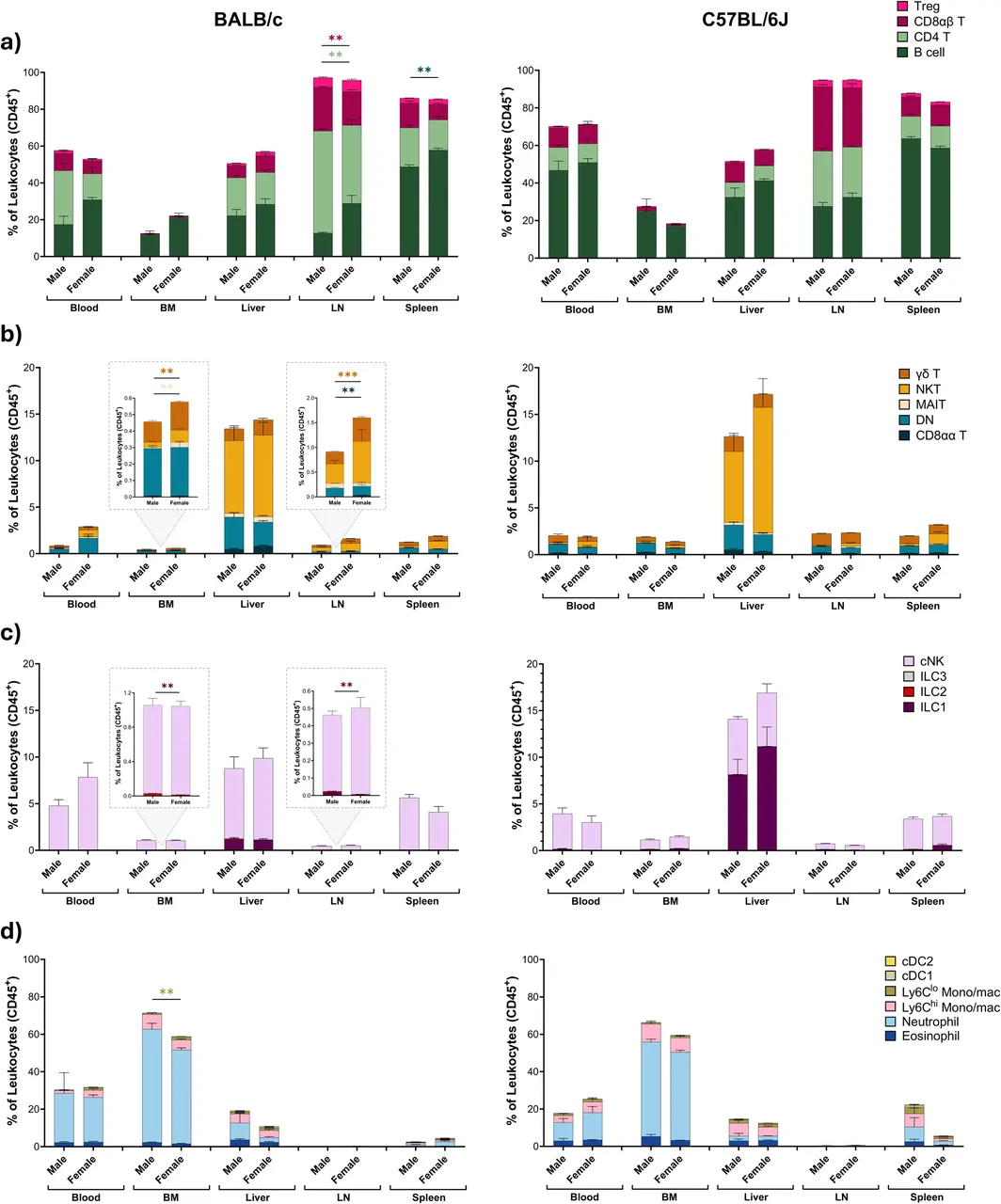
Sex‐associated differences in leukocyte composition are modest across murine tissues. Proportion of (a) conventional lymphoid, (b) unconventional T, (c) innate lymphoid (ILC), and (d) myeloid, cell subsets in the blood, bone marrow (BM), liver, lymph nodes (LN), and spleen of male (M) and female (F) BALB/c and C57BL/6J mice. Flow cytometry data were acquired across multiple days with pre‐acquisition quality controls to ensure consistency across runs. Differences were identified using unpaired or Welch's *t*‐tests, or Mann–Whitney tests, using the two‐stage Benjamini, Krieger, and Yekutieli procedure to control the false discovery rate. Data are presented as mean + SEM. A/B: C57BL/6J M, *n* = 5 (LN, *n* = 4); C57BL/6J F, *n* = 3; BALB/c M, *n* = 4; BALB/c F, *n* = 4 (LN, *n* = 3). C: C57BL/6J M, *n* = 5 (liver and LN, *n* = 4); C57BL/6J F, *n* = 3 (liver, *n* = 4); BALB/c M, *n* = 4; BALB/c F, *n* = 4 (LN, *n* = 3). D: C57BL/6J M, *n* = 5 (blood and LN, *n* = 3); C57BL/6J F, *n* = 3; BALB/c M, *n* = 4; BALB/c F, *n* = 4 (LN, *n* = 3). *p < 0.05, **p < 0.01, and ***p < 0.001. Color of symbols indicates the relevant population comparison. Treg, regulatory T cell; NKT, natural killer T cell; MAIT, mucosal‐associated invariant T cell; DN, TCRβ^+^CD4^−^CD8^−^double negative T cell; cNK, conventional NK cell; cDC, conventional dendritic cell; mono/mac, monocyte/macrophage.

The frequencies of CD4 and CD8 T cell memory phenotypes were also largely comparable between sexes, with the most pronounced difference being an enrichment of naïve CD4 and CD8 T cells in the blood of male compared to female BALB/c mice (Figure S8).

## 4 Discussion

Variation in immunophenotypes between mouse models can alter the outcome and interpretation of immunological studies, yet comprehensive sex‐ and strain‐specific comparisons across tissues remain limited. In this study, we used flow cytometry to immunophenotype 19 leukocyte subsets in the blood, BM, liver, LN, and spleen of seven commonly used laboratory mouse strains to extend established observations from peripheral blood to additional tissues frequently examined in immunological research.

BALB‐derived strains (Balb.b, BALB/c, and BALB/c Ly5.1) shared a broadly similar immune profile across tissues, characterized by reduced B cell levels and enrichment of CD4 T cells, Tregs, neutrophils, and cNK cells. This profile indicates the immune environment of BALB‐derived strains is skewed toward helper T cell‐mediated immune responses and neutrophil and cNK cell immunity. This is consistent with evidence of BALB/c mice mounting Th2‐biased immune responses to injury, parasites, and antigens, and robust early innate responses driven by pro‐inflammatory cytokines [24, 25, 26, 27, 28]. For example, liver injury in BALB/c mice was found to result in severe fibrosis, whereas for the same injury in C57BL/6 mice, fibrosis was relatively minimal [25]. Therefore, BALB‐derived mice are suited to study allergy and early inflammatory responses and may be particularly useful for exploring how CD4^+^ T cells coordinate with innate effector cells to initiate and control Th2 immune responses. While overall T cell frequencies were similar between BALB‐derived strains, differences in T cell memory phenotypes were evident, with Balb.b mice displaying reduced naïve and increased memory (CD4 T_cm_ and CD8 T_em_) populations. This greater pool of antigen‐experienced T cells at baseline may make this strain less suitable for functional studies where T cell differentiation or memory responses are important.

C57BL/6J‐derived strains (C57BL/6J and C57BL/6J Ly5.1) also had comparable immune profiles, enriched for CD8αβ and CD8αα T cells, ILC1s, and cDCs. Together, these immune subsets are indicative of strong cytotoxic effector function and efficient antigen surveillance, aligning with consistent reports of C57BL/6J mice as Th1‐biased, with strong IFNγ‐mediated responses and effective CD8^+^ T cell‐mediated antigen clearance in response to infection and microbial ligands [29, 30, 31, 32]. The enrichment of CD8 T_cm_ cells alongside reduced CD8 T_n_ cells in these strains further suggests a baseline T cell phenotype associated with greater readiness for cytotoxic responses. This evidence supports the use of C57BL/6J‐derived mice in viral, tumor, and allograft rejection models where antigen‐specific immune responses are critical to outcomes. This is particularly relevant for studies of hepatic infection, tumor metastasis, and liver transplantation, given the marked enrichment of hepatic ILC1s [33, 34, 35].

Ly5.1 congenic strains theoretically share > 99% of their genetics with their parental strain and so are often used interchangeably [36]. While broadly similar, we did observe significant differences in immune composition between wild‐type and congenic strains of both BALB/c and C57BL/6J mice. These differences likely result from a combination of retained genetics, epigenetic drift, breeding effects, and environmental factors [37, 38, 39], emphasizing the value of substrain‐matched controls, particularly when investigating rarer immune phenotypes.

The F1 mice presented here were generated from crossing wildtype BALB/c and C57BL/6J mice, and therefore carry alleles from both parental strains. We found that for some cell subsets (e.g., ILC1s and neutrophils), the frequencies in F1 mice fell between those of either parental strain. However, these mice did not always exhibit intermediate immune phenotypes, and several leukocyte populations such as B cells and conventional T cells more closely aligned with C57BL/6J proportions. This is consistent with findings from T cell regulation and infection‐related studies, where F1 mice demonstrated both averaged and biased inheritance of traits opposed to uniform inheritance patterns [40, 41]. This is important to consider when hybrid mice are selected with the expectation of a balanced immune response, as is common in infection, immunogenetic, and transplantation studies.

The immune profile of DBA/2 J mice was distinct from mice of both BALB and C57BL/6J backgrounds, primarily through elevated γδ T cell frequencies in the liver, blood, LN, and spleen. Although there is limited information regarding the immune composition of naïve DBA/2 J mice, this strain is reported to exhibit strong inflammatory potential with elevation of myeloid cells and hematopoietic progenitors [9, 42, 43, 44, 45]. While myeloid cells were not enriched in this study, the elevated γδ T cell representation observed here is consistent with an innate‐skewed immune environment, given the capacity of γδ T cells to rapidly produce cytokines that recruit neutrophils and macrophages and initiate rapid responses at epithelial barriers [46, 47]. Collectively, these findings support the suitability of DBA/2 J mice in studies of epithelial defense and innate‐like T cell function. The relatively low levels of NKT cells in these tissues may further limit NKT‐mediated interactions during early immune activation, enabling clearer resolution of γδ T cell‐driven pathways.

An additional difference observed across strains was the variation in neutrophil CD11b and Ly6G expression. Few studies directly compare CD11b or Ly6G MFI between naïve mouse strains; however, their expression is known to vary according to neutrophil maturation state, activation status, and tissue localization [48, 49, 50]. For example, Ly6G expression has been found to increase as mouse neutrophils mature, with Ly6G^int^ cells representing immature neutrophils and Ly6G^hi^ cells representing mature neutrophils in both steady‐state and disease conditions [49, 50]. Further, colonic and oral cavity neutrophils were shown to have up to sixfold greater baseline CD11b expression compared with six other mouse tissues [48]. The strain‐dependent differences in MFI observed in this study therefore likely reflect homeostatic differences in neutrophil maturation states. This highlights that commonly used immunophenotypic markers are not always uniformly expressed by specific cell types.

Alongside strain, sex exerted a modest influence on immune composition across tissues. Our observations align with previous flow cytometric studies of peripheral blood, spleen, and BM, in which strain effects typically exceeded sex‐associated differences [7, 12]. Reported sex‐associated differences generally show that female mice mount stronger adaptive immune responses, whereas males display a relative myeloid bias following immune challenge, including in C57BL/6J, BALB/cJ, and RAG2^−/−^ strains [51, 52, 53]. Consistent with this, we observed subtle trends toward higher B cell and NKT cell frequencies in female mice across several tissues compared to increased neutrophil proportions in males, although most differences did not reach statistical significance, likely reflecting limited sample size. However, we also detected increased frequencies of CD8αβ and CD4 T cells in male BALB/c tissues, contrasting with the expected female‐biased lymphoid profile. This discrepancy may reflect strain‐specific variation under naïve conditions, or be related to factors such as age or cage‐associated effects. While overall our findings indicate that the influence of sex is comparatively minor relative to strain, these differences may become functionally relevant under certain experimental conditions.

In addition to sex and strain, immune composition is shaped by a number of biological and environmental factors such as age, handling practices, and microbiome variation [39, 54, 55]. The mice in this study spanned a broad adult age range (13–22 weeks) due to their inclusion in concurrent groups across related studies. While mouse immune systems are generally considered mature across this range, ongoing immunological maturation and early aging during this period may contribute to variability in immune cell frequencies and activation states [55]. Further, while standard housing conditions were uniform, cage‐associated effects may have occurred with each cage containing a specific microenvironment. These variables should therefore be considered when interpreting subtle differences and underscore that baseline immune profiles reflect a combination of genetic and environmental influences.

Importantly, this resource establishes a foundation for the development of a comprehensive reference atlas of baseline murine immune composition, supporting more informed model selection and experimental design. Incorporating the 3Rs (replacement, reduction, and refinement) into the design and conduct of animal research has become fundamental to ethical experimental practice [56]. This dataset can be used to more closely align animal models with the research question being addressed while minimizing the potential confounding effects of sex and strain, thereby reducing animal use and enabling more robust comparisons. Future work could expand this resource by incorporating additional mouse strains, tissues, and more deeply resolved immune populations, such as B cell lineage subsets, while applying contemporary flow cytometric immunophenotyping and gating strategies aligned with consensus classifications to better define, for example, monocyte and DC subpopulations [57]. An additional consideration is strain‐ and sex‐based variation in organ cellularity or size. Future studies incorporating normalization of organ weight and body weight will be important to determine whether proportional changes correspond to alterations in absolute cellularity. The absolute cell numbers reported here were calculated from total cell counts following nonenzymatic tissue processing, and may therefore differ from counts obtained using alternative tissue processing methods.

Overall, this study provides a reference for tissue‐specific immune composition across multiple mouse strains, tissues, and sexes. Our findings highlight the importance of intentional strain and sex selection when using mouse models in immunological studies, and the value of defining baseline immune profiles to enable meaningful comparisons following experimental intervention. Continuing to characterize and transparently report sources of biological variability will facilitate more accurate interpretation of immune phenotypes, strengthen reproducibility within and between studies, and minimize the risk of conflating biological variability with true experimental effects.

## Author Contributions

**Ella Trolio:** conceptualization, data curation, investigation, formal analysis, writing – original draft, writing – review and editing, visualization. **Sarah Dart:** conceptualization, methodology, investigation, writing – review and editing, data curation. **Liu Liu:** investigation, data curation. **Michaela Lucas:** conceptualization, funding acquisition, supervision. **Amy Prosser:** conceptualization, methodology, investigation, data curation, writing – review and editing, funding acquisition, supervision.

## Funding

This work was supported by the Charlies Foundation for Research, DPG24‐25_05. Department of Health, Government of Western Australia.

## Ethics Statement

All experiments were conducted in compliance with the Australian Code for the Care and Use of Animals for Scientific Purposes and approved by the University of Western Australia and Harry Perkins Institute for Medical Research Animal Ethics Committees (protocols RA/3/100/1568, AE173, AE279 and AE372).

## Conflicts of Interest

The authors declare no conflicts of interest.

## Supporting information


**Figure S1:** Lymphocyte subset gating strategy. Gating strategy to identify (a) B cells, TCRβ^+^ T cells, γδ T cells, natural killer T (NKT) NKT cells, mucosal‐associated invariant T (MAIT) cells, TCRβ^+^CD4^−^CD8^−^ double negative (DN) T cells, CD4 T cells, regulatory T cells (Tregs), CD8αβ T cells, and CD8α^+^β^−^ (CD8αα) T cells, and (b) naïve (T_n_), central memory (T_cm_) and effector memory (T_em_) CD4 and CD8 T cell subsets. Representative flow cytometry plots from mouse spleen.

**Figure S2:** Innate lymphoid cell (ILC) gating strategy. Gating strategy to identify conventional natural killer (cNK), and ILC Types 1, 2, and 3. Representative flow cytometry plots from mouse spleen.

**Figure S3:** Myeloid cell subset gating strategy. Gating strategy to identify neutrophils, eosinophils, Ly6C^hi^ and Ly6C^lo^ monocytes/macrophages, and Type 1 and Type 2 conventional dendritic cells (cDCs). Representative flow cytometry plots from mouse spleen.

**Figure S4:** Total T cell frequencies vary largely between tissues, with comparatively modest differences between mouse strains. Proportion of total TCRβ^+^ T cells of CD45^+^ leukocytes in the blood, bone marrow (BM), liver, lymph nodes (LN) and spleen. Flow cytometry data were acquired across multiple days with pre‐acquisition quality controls to ensure consistency across runs. Differences were identified using Ordinary One‐Way or Welch's ANOVA, or Kruskal–Wallis tests, followed by Tukey's, Dunnett's, or Dunn's multiple comparisons corrections. Data are presented as mean + SEM. Balb.b, *n* = 5; BALB/c, *n* = 4; BALB/c Ly5.1, *n* = 6 (LN, *n* = 3); C57BL/6J, *n* = 5 (LN, *n* = 4); C57BL/6J Ly5.1, *n* = 8 (LN, *n* = 3); F1 (BALB/c:B6J), *n* = 5 (LN, *n* = 1); and DBA/2 J, *n* = 5. *adj. *p* < 0.05, ** adj. *p* < 0.01, and *** adj. *p* < 0.001.

**Figure S5:** Pairwise comparisons identify distinct strain‐specific differences in naïve and memory CD4 and CD8 T cell subsets. Heatmap of pairwise differences in the proportion of naïve (T_n_), central memory (T_cm_) and effector memory (T_em_) subsets within CD69^−^ (a) CD4 and (b) CD8 T cells in the blood, bone marrow (BM), liver, lymph nodes (LN), and spleen. Values represent magnitude of change in frequency. Positive values indicate enrichment and negative values indicate reduction in the bracket title strain relative to the bracketed strain. Significant differences were identified using Ordinary One‐Way or Welch's ANOVA, or Kruskal–Wallis tests, followed by Tukey's, Dunnett's, or Dunn's multiple comparisons corrections. Balb.b, *n* = 5; BALB/c, *n* = 4; BALB/c Ly5.1, *n* = 6 (LN, *n* = 3); C57BL/6J, *n* = 5 (LN, *n* = 4); C57BL/6J Ly5.1, *n* = 8 (LN, *n* = 3); F1 (BALB/c:B6J), *n* = 5 (LN, *n* = 1); and DBA/2 J, *n* = 5. Unshaded cells indicate nonsignificant comparisons (adj. *p* > 0.05).

**Figure S6:** Neutrophil populations show strain‐ and tissue‐specific variation in expression of key surface proteins. Representative flow cytometry plots showing neutrophil populations in the blood, bone marrow (BM), liver, lymph nodes (LN), and spleen. C57BL/6J Ly5.1 LN samples excluded due to use of an alternative fluorophore (SB600) following antibody discontinuation.

**Figure S7:** Neutrophil CD11b and Ly6G expression exhibits strain‐ and tissue‐specific variation. Median fluorescence intensity (MFI) of (a) CD11b and (b) Ly6G on neutrophils in the blood, bone marrow (BM), liver, lymph nodes (LN), and spleen. Flow cytometry data were acquired across multiple days with pre‐acquisition quality controls to ensure consistency across runs. Differences were identified using Ordinary One‐Way or Welch's ANOVA, or Kruskal–Wallis tests, followed by Tukey's, Dunnett's, or Dunn's multiple comparisons corrections. Data are presented as mean + SEM. Balb.b, *n* = 5; BALB/c, *n* = 4; BALB/c Ly5.1, *n* = 6 (LN, *n* = 3); C57BL/6J, *n* = 5 (LN and blood, *n* = 3); C57BL/6J Ly5.1, *n* = 8 (LN, *n* = 3); F1 (BALB/c:B6J), *n* = 3 (LN, *n* = 1); and DBA/2 J, *n* = 5 (spleen, *n* = 4). *adj. *p* < 0.05, **adj. *p* < 0.01, and ***adj. *p* < 0.001. C57BL/6J Ly5.1 LN samples excluded from CD11b analysis due to use of an alternative fluorophore (SB600) following antibody discontinuation.

**Figure S8:** Sex‐associated differences in T cell memory phenotypes are modest across murine tissues. Percentage (%) of naïve (T_n_), central memory (T_cm_), and effector memory (T_em_) T cell subsets of CD69^−^ CD4 and CD8 T cells in the blood, bone marrow (BM), liver, lymph nodes (LN), and spleen of male and female BALB/c and C57BL/6J mice. Flow cytometry data were acquired across multiple days with pre‐acquisition quality controls to ensure consistency across runs. Differences were identified using unpaired or Welch's *t*‐tests, or Mann–Whitney tests, using the two‐stage Benjamini, Krieger, and Yekutieli procedure to control the false discovery rate. Data are presented as mean. A/B: C57BL/6J M, *n* = 5 (LN, *n* = 4); C57BL/6J F, *n* = 3; BALB/c M, *n* = 4; BALB/c F, *n* = 4 (LN, *n* = 3). C: C57BL/6J M, *n* = 5 (liver and LN, *n* = 4); C57BL/6J F, *n* = 3 (liver, *n* = 4); BALB/c M, *n* = 4; BALB/c F, *n* = 4 (LN, *n* = 3). D: C57BL/6J M, *n* = 5 (blood and LN, *n* = 3); C57BL/6J F, *n* = 3; BALB/c M, *n* = 4; BALB/c F, *n* = 4 (LN, *n* = 3). *p < 0.05, **p < 0.01, and ***p < 0.001.

**Table S1:** Mean absolute numbers of leukocyte subsets in the blood (cells/mL). Sample sizes (n) are as specified in main text Figures 1, 2, 4, 5, 6.
**Table S2:** Mean absolute numbers of leukocyte subsets in the bone marrow. Sample sizes (n) are as specified in main text Figures 1, 2, 4, 5, 6.
**Table S3:** Mean absolute numbers of leukocyte subsets in the liver. Sample sizes (n) are as specified in main text Figures 1, 2, 4, 5, 6.
**Table S4:** Mean absolute numbers of leukocyte subsets in the abdominal lymph nodes (~10–12 pooled). Sample sizes (n) are as specified in main text Figures 1, 2, 4, 5, 6.
**Table S5:** Mean absolute numbers of leukocyte subsets in the spleen. Sample sizes (n) are as specified in main text Figures 1, 2, 4, 5, 6.

## Data Availability

The data that support the findings of this study are available from the corresponding author upon reasonable request.
