# Supplementary material for: The Leukocyte Landscape Across Mouse Strains and Tissues
Source: Immunol Cell Biol. 2026 Oct 8;104(10):1019–34. doi: 10.1111/imcb.70168 (PMC13743906; doi:10.1111/imcb.70168)
Supplement: Supplementary file 1 [file IMCB-104-1019-s004.docx]

**Supporting Information**

**Figure S1. Lymphocyte subset gating strategy.** Gating strategy to identify **a)** B cells, TCRβ^+^ T cells, γδ T cells, natural killer T (NKT) NKT cells, mucosal-associated invariant T (MAIT) cells, TCRβ^+^CD4^–^CD8^–^ double negative (DN) T cells, CD4 T cells, regulatory T cells (Tregs), CD8αβ T cells, and CD8α^+^β^–^ (CD8αα) T cells, and **b)** naïve (T_n_), central memory (T_cm_) and effector memory (T_em_) CD4 and CD8 T cell subsets. Representative flow cytometry plots from mouse spleen.

**Figure S2. Innate lymphoid cell (ILC) gating strategy.** Gating strategy to identify conventional natural killer (cNK), and innate lymphoid cells (ILC) types 1, 2, and 3. Representative flow cytometry plots from mouse spleen.

**Figure S3. Myeloid cell subset gating strategy.** Gating strategy to identify neutrophils, eosinophils, Ly6C^hi^ and Ly6C^lo^ monocytes/macrophages, and type 1 and type 2 conventional dendritic cells (cDCs). Representative flow cytometry plots from mouse spleen.

**Figure S4. Total T cell frequencies vary largely between tissues, with comparatively modest differences between mouse strains.** Proportion of total TCRβ^+^ T cells of CD45^+^ leukocytes in the blood, bone marrow (BM), liver, lymph nodes (LN) and spleen. Flow cytometry data were acquired across multiple days with pre-acquisition quality controls to ensure consistency across runs. Differences were identified using Ordinary One-Way or Welch’s ANOVA, or Kruskal–Wallis tests, followed by Tukey’s, Dunnett’s, or Dunn’s multiple comparisons corrections. Data are presented as mean + SEM. Balb.b, n=5; BALB/c, n=4; BALB/c Ly5.1, n=6 (LN, n=3); C57BL/6J, n=5 (LN, n=4); C57BL/6J Ly5.1, n=8 (LN, n=3); F1 (BALB/c:B6J), n=5 (LN, n=1); and DBA/2J, n=5. *adj. p<0.05, ** adj. p<0.01, *** adj. p<0.001.

**Figure S5. Pairwise comparisons identify distinct strain-specific differences in naïve and memory CD4 and CD8 T cell subsets.** Heatmap of pairwise differences in the proportion of naïve (T_n_), central memory (T_cm_) and effector memory (T_em_) subsets within CD69⁻ **a)** CD4 and **b)** CD8 T cells in the blood, bone marrow (BM), liver, lymph nodes (LN) and spleen. Values represent magnitude of change in frequency. Positive values indicate enrichment and negative values indicate reduction in the bracket title strain relative to the bracketed strain. Significant differences were identified using Ordinary One-Way or Welch’s ANOVA, or Kruskal–Wallis tests, followed by Tukey’s, Dunnett’s, or Dunn’s multiple comparisons corrections. Balb.b, n=5; BALB/c, n=4; BALB/c Ly5.1, n=6 (LN, n=3); C57BL/6J, n=5 (LN, n=4); C57BL/6J Ly5.1, n=8 (LN, n=3); F1 (BALB/c:B6J), n=5 (LN, n=1); and DBA/2J, n=5. Unshaded cells indicate non-significant comparisons (adj. p>0.05).

**Figure S6. Neutrophil populations show strain- and tissue-specific variation in expression of key surface proteins.** Representative flow cytometry plots showing neutrophil populations in the blood, bone marrow (BM), liver, lymph nodes (LN) and spleen. C57BL/6J Ly5.1 LN samples excluded due to use of an alternative fluorophore (SB600) following antibody discontinuation.

**Figure S7. Neutrophil CD11b and Ly6G expression exhibits strain- and tissue-specific variation.** Median fluorescence intensity (MFI) of **a)** CD11b and **b)** Ly6G on neutrophils in the blood, bone marrow (BM), liver, lymph nodes (LN) and spleen. Flow cytometry data were acquired across multiple days with pre-acquisition quality controls to ensure consistency across runs. Differences were identified using Ordinary One-Way or Welch’s ANOVA, or Kruskal–Wallis tests, followed by Tukey’s, Dunnett’s, or Dunn’s multiple comparisons corrections. Data are presented as mean + SEM. Balb.b, n=5; BALB/c, n=4; BALB/c Ly5.1, n=6 (LN, n=3); C57BL/6J, n=5 (LN and blood, n=3); C57BL/6J Ly5.1, n=8 (LN, n=3); F1 (BALB/c:B6J), n=3 (LN, n=1); and DBA/2J, n=5 (spleen, n=4). *adj. p<0.05, **adj. p<0.01, ***adj. p<0.001. C57BL/6J Ly5.1 LN samples excluded from CD11b analysis due to use of an alternative fluorophore (SB600) following antibody discontinuation.

**Figure S8. Sex-associated differences in T cell memory phenotypes are modest across murine tissues.** Percentage (%) of naïve (T_n_), central memory (T_cm_) and effector memory (T_em_) T cell subsets of CD69^–^ CD4 and CD8 T cells in the blood, bone marrow (BM), liver, lymph nodes (LN) and spleen of male and female BALB/c and C57BL/6J mice. Flow cytometry data were acquired across multiple days with pre-acquisition quality controls to ensure consistency across runs. Differences were identified using unpaired or Welch’s t-tests, or Mann-Whitney tests, using the two-stage Benjamini, Krieger, & Yekutieli procedure to control the false discovery rate. Data are presented as mean. A/B: C57BL/6J M, n=5 (LN, n=4); C57BL/6J F, n=3; BALB/c M, n=4; BALB/c F, n=4 (LN, n=3). C: C57BL/6J M, n=5 (liver and LN, n=4); C57BL/6J F, n=3 (liver, n=4); BALB/c M, n=4; BALB/c F, n=4 (LN, n=3). D: C57BL/6J M, n=5 (blood and LN, n=3); C57BL/6J F, n=3; BALB/c M, n=4; BALB/c F, n=4 (LN, n=3). *p<0.05, **p<0.01, ***p<0.001.

**Table S1. Mean absolute numbers of leukocyte subsets in the blood (cells/mL).** Sample sizes (n) are as specified in main text Figures 1, 2, 4, 5 and 6.

|  | **Male** | | | | | | | **Female** | |
| --- | --- | --- | --- | --- | --- | --- | --- | --- | --- |
|  | **Balb.b** | **BALB/c** | **BALB/c Ly5.1** | **C57BL/6J** | **C57BL/6J Ly5.1** | **F1** | **DBA/2J** | **BALB/c** | **C57BL/6J** |
| **B cell** | 7.88E+04 | 2.17E+05 | 4.36E+05 | 1.60E+06 | 5.10E+05 | 5.97E+05 | 6.92E+05 | 4.77E+04 | 1.45E+05 |
| **CD4 T** | 1.70E+05 | 4.09E+05 | 4.24E+05 | 1.87E+05 | 1.23E+05 | 2.06E+05 | 3.81E+05 | 1.27E+04 | 2.92E+04 |
| **CD8αβ T** | 3.92E+04 | 1.28E+05 | 2.04E+05 | 1.68E+05 | 1.24E+05 | 1.71E+05 | 1.48E+05 | 7.31E+03 | 2.85E+04 |
| **Treg** | 1.50E+04 | 2.37E+04 | 4.18E+04 | 1.36E+04 | 9.45E+03 | 1.02E+04 | 2.46E+04 | 1.11E+03 | 9.73E+02 |
| **CD8αα T** | 2.21E+02 | 8.80E+01 | 2.65E+02 | 4.93E+03 | 9.18E+02 | 5.16E+02 | 2.70E+02 | 3.37E+01 | 2.37E+02 |
| **DN T** | 1.48E+03 | 7.91E+03 | 1.75E+04 | 2.66E+04 | 6.90E+03 | 3.20E+03 | 2.01E+04 | 3.33E+03 | 1.70E+03 |
| **MAIT** | 2.28E+02 | 1.40E+03 | 1.53E+03 | 5.08E+02 | 7.20E+02 | 3.41E+02 | 2.33E+02 | 1.12E+02 | 6.44E+01 |
| **NKT** | 6.66E+03 | 8.71E+02 | 3.07E+03 | 3.94E+03 | 1.20E+03 | 4.22E+03 | 1.90E+03 | 5.12E+02 | 1.15E+03 |
| **γδT** | 3.76E+03 | 2.60E+03 | 1.01E+04 | 2.06E+04 | 6.49E+03 | 2.03E+03 | 1.47E+04 | 5.00E+02 | 1.40E+03 |
| **ILC1** | 1.40E+02 | 3.54E+02 | 1.80E+02 | 8.19E+03 | 1.03E+03 | 2.19E+02 | 3.05E+02 | 2.78E+01 | 1.04E+02 |
| **ILC2** | 6.67E+01 | 5.57E+01 | 1.02E+00 | 7.14E+00 | 0.00E+00 | 1.35E+02 | 7.18E+01 | 7.16E-01 | 0.00E+00 |
| **ILC3** | 4.96E+01 | 0.00E+00 | 8.72E+01 | 4.05E+02 | 0.00E+00 | 1.22E+02 | 0.00E+00 | 1.29E+00 | 2.41E+00 |
| **cNK** | 4.72E+04 | 7.03E+04 | 3.40E+04 | 9.04E+04 | 2.47E+04 | 5.43E+04 | 3.79E+04 | 1.19E+04 | 7.53E+03 |
| **Eosinophil** | 2.90E+04 | 2.71E+04 | 5.82E+04 | 3.60E+04 | 4.59E+04 | 1.65E+04 | 4.45E+04 | 3.27E+03 | 9.97E+03 |
| **Neutrophil** | 4.13E+05 | 1.44E+05 | 8.57E+05 | 1.04E+05 | 2.31E+04 | 1.23E+05 | 2.26E+05 | 3.96E+04 | 4.33E+04 |
| **Ly6C^hi^ Mono/mac** | 8.94E+04 | 1.32E+04 | 1.11E+05 | 4.15E+04 | 5.62E+04 | 1.49E+04 | 6.05E+04 | 8.85E+03 | 1.67E+04 |
| **Ly6C^lo^ Mono/mac** | 9.84E+03 | 5.79E+03 | 3.49E+04 | 9.70E+03 | 2.65E+04 | 6.79E+03 | 3.57E+04 | 2.49E+03 | 4.10E+03 |
| **cDC1** | 6.45E+01 | 1.92E+02 | 2.64E+02 | 1.81E+02 | 1.23E+02 | 5.40E+01 | 4.96E+02 | 2.55E+01 | 7.04E+00 |
| **cDC2** | 1.04E+02 | 2.83E+02 | 7.96E+02 | 4.40E+02 | 5.37E+02 | 2.86E+02 | 1.10E+03 | 4.65E+01 | 7.53E+01 |

*Treg, regulatory T cell; DN, TCRβ^+^CD4^–^CD8^–^double negative T cell; MAIT, mucosal-associated invariant T cell; NKT, natural killer T cell; ILC, innate lymphoid cell; cNK, conventional NK cell; mono/mac, monocyte/macrophage; cDC, conventional dendritic cell.*

**Table S2. Mean absolute numbers of leukocyte subsets in the bone marrow.** Sample sizes (n) are as specified in main text Figures 1, 2, 4, 5 and 6.

|  | **Male** | | | | | | | **Female** | |
| --- | --- | --- | --- | --- | --- | --- | --- | --- | --- |
|  | **Balb.b** | **BALB/c** | **BALB/c Ly5.1** | **C57BL/6J** | **C57BL/6J Ly5.1** | **F1** | **DBA/2J** | **BALB/c** | **C57BL/6J** |
| **B cell** | 2.31E+06 | 1.28E+06 | 1.09E+06 | 1.78E+06 | 5.04E+06 | 5.07E+06 | 2.85E+06 | 1.68E+06 | 2.35E+06 |
| **CD4 T** | 3.83E+04 | 2.93E+04 | 2.40E+04 | 2.62E+04 | 8.02E+04 | 3.34E+04 | 1.83E+04 | 2.39E+04 | 4.28E+04 |
| **CD8αβ T** | 6.08E+04 | 6.76E+04 | 3.26E+04 | 1.39E+05 | 2.41E+05 | 1.46E+05 | 3.36E+04 | 5.31E+04 | 1.31E+05 |
| **Treg** | 3.58E+04 | 2.92E+04 | 1.87E+04 | 3.39E+04 | 5.11E+04 | 4.15E+04 | 3.35E+04 | 2.53E+04 | 2.57E+04 |
| **CD8αα T** | 2.01E+03 | 8.23E+02 | 7.63E+02 | 1.68E+04 | 2.03E+04 | 2.62E+03 | 9.86E+02 | 5.79E+02 | 1.48E+04 |
| **DN T** | 1.92E+04 | 2.97E+04 | 1.81E+04 | 7.21E+04 | 1.09E+05 | 3.19E+04 | 3.78E+04 | 2.49E+04 | 8.27E+04 |
| **MAIT** | 2.67E+03 | 7.33E+02 | 9.18E+02 | 7.49E+02 | 2.47E+03 | 1.70E+03 | 5.45E+02 | 2.44E+03 | 3.38E+03 |
| **NKT** | 1.54E+04 | 3.13E+03 | 2.83E+03 | 1.13E+04 | 2.79E+04 | 1.73E+04 | 5.73E+03 | 6.02E+03 | 3.74E+04 |
| **γδT** | 2.92E+04 | 1.32E+04 | 1.49E+04 | 3.19E+04 | 6.06E+04 | 2.90E+04 | 2.88E+04 | 1.40E+04 | 5.53E+04 |
| **ILC1** | 1.97E+03 | 9.75E+02 | 9.89E+02 | 1.21E+04 | 2.99E+04 | 2.63E+03 | 4.15E+03 | 4.12E+02 | 3.17E+04 |
| **ILC2** | 3.25E+03 | 2.78E+03 | 7.66E+02 | 4.01E+03 | 2.22E+03 | 1.50E+03 | 1.50E+04 | 9.54E+02 | 1.35E+03 |
| **ILC3** | 4.68E+03 | 2.33E+02 | 2.16E+02 | 3.62E+02 | 1.03E+02 | 1.98E+02 | 2.72E+03 | 2.70E+02 | 1.38E+02 |
| **cNK** | 1.99E+05 | 1.32E+05 | 5.60E+04 | 1.16E+05 | 2.22E+05 | 1.12E+05 | 1.69E+05 | 1.19E+05 | 1.95E+05 |
| **Eosinophil** | 5.21E+05 | 2.54E+05 | 1.22E+05 | 4.46E+05 | 5.40E+05 | 2.56E+05 | 4.19E+05 | 1.28E+05 | 4.69E+05 |
| **Neutrophil** | 1.21E+07 | 6.83E+06 | 3.18E+06 | 5.04E+06 | 5.22E+06 | 7.20E+06 | 5.68E+06 | 4.01E+06 | 6.68E+06 |
| **Ly6C^hi^ Mono/mac** | 1.46E+06 | 8.69E+05 | 4.44E+05 | 8.57E+05 | 1.43E+06 | 8.53E+05 | 1.12E+06 | 4.07E+05 | 1.06E+06 |
| **Ly6C^lo^ Mono/mac** | 1.36E+05 | 7.77E+04 | 3.31E+04 | 6.86E+04 | 2.56E+05 | 5.50E+04 | 9.64E+04 | 1.42E+05 | 1.59E+05 |
| **cDC1** | 1.80E+03 | 2.37E+03 | 1.46E+03 | 2.03E+03 | 8.93E+03 | 5.68E+02 | 2.90E+03 | 3.48E+03 | 4.59E+03 |
| **cDC2** | 4.86E+03 | 2.98E+03 | 1.87E+03 | 2.87E+03 | 2.50E+04 | 2.44E+03 | 5.08E+03 | 3.52E+03 | 9.00E+03 |

*Treg, regulatory T cell; DN, TCRβ^+^CD4^–^CD8^–^double negative T cell; MAIT, mucosal-associated invariant T cell; NKT, natural killer T cell; ILC, innate lymphoid cell; cNK, conventional NK cell; mono/mac, monocyte/macrophage; cDC, conventional dendritic cell.*

**Table S3. Mean absolute numbers of leukocyte subsets in the liver.** Sample sizes (n) are as specified in main text Figures 1, 2, 4, 5 and 6.

|  | **Male** | | | | | | | **Female** | |
| --- | --- | --- | --- | --- | --- | --- | --- | --- | --- |
|  | **Balb.b** | **BALB/c** | **BALB/c Ly5.1** | **C57BL/6J** | **C57BL/6J Ly5.1** | **F1** | **DBA/2J** | **BALB/c** | **C57BL/6J** |
| **B cell** | 8.12E+03 | 1.12E+05 | 2.74E+05 | 1.39E+05 | 4.30E+05 | 1.80E+05 | 9.26E+04 | 4.34E+05 | 1.17E+06 |
| **CD4 T** | 2.42E+04 | 1.03E+05 | 1.47E+05 | 3.67E+04 | 9.15E+04 | 6.39E+04 | 4.34E+04 | 2.66E+05 | 2.23E+05 |
| **CD8αβ T** | 7.07E+03 | 3.11E+04 | 8.92E+04 | 4.67E+04 | 1.25E+05 | 5.55E+04 | 2.16E+04 | 1.35E+05 | 2.21E+05 |
| **Treg** | 3.58E+03 | 7.40E+03 | 1.87E+04 | 4.60E+03 | 9.24E+03 | 4.70E+03 | 6.59E+03 | 3.26E+04 | 2.37E+04 |
| **CD8αα T** | 3.06E+02 | 2.01E+03 | 3.02E+03 | 2.85E+03 | 1.00E+04 | 8.34E+02 | 2.74E+02 | 1.20E+04 | 7.94E+03 |
| **DN T** | 1.48E+03 | 1.74E+04 | 2.73E+04 | 1.23E+04 | 3.30E+04 | 4.26E+03 | 3.68E+03 | 3.90E+04 | 5.33E+04 |
| **MAIT** | 4.34E+02 | 2.09E+03 | 5.12E+03 | 1.30E+03 | 2.45E+03 | 5.12E+02 | 3.07E+02 | 9.72E+03 | 5.16E+03 |
| **NKT** | 4.92E+03 | 4.09E+04 | 5.94E+04 | 3.24E+04 | 1.08E+05 | 2.56E+04 | 4.10E+03 | 1.42E+05 | 3.68E+05 |
| **γδT** | 2.69E+03 | 5.68E+03 | 1.83E+04 | 7.34E+03 | 1.71E+04 | 4.50E+03 | 1.08E+04 | 2.42E+04 | 3.97E+04 |
| **ILC1** | 3.46E+02 | 5.11E+03 | 6.99E+03 | 4.03E+04 | 1.10E+05 | 1.50E+04 | 1.65E+03 | 1.69E+04 | 3.06E+05 |
| **ILC2** | 5.42E+01 | 4.55E+02 | 3.09E+02 | 1.24E+02 | 1.15E+02 | 4.74E+01 | 2.30E+02 | 2.76E+02 | 2.04E+02 |
| **ILC3** | 3.38E+01 | 6.34E+01 | 3.00E+02 | 1.46E+02 | 1.68E+01 | 8.19E+00 | 2.89E+01 | 7.58E+01 | 7.15E+01 |
| **cNK** | 1.58E+04 | 3.63E+04 | 4.37E+04 | 3.13E+04 | 7.83E+04 | 4.80E+04 | 2.36E+04 | 1.27E+05 | 1.32E+05 |
| **Eosinophil** | 6.04E+03 | 1.63E+04 | 4.69E+04 | 2.66E+04 | 4.00E+04 | 1.57E+04 | 1.01E+04 | 3.30E+04 | 7.20E+04 |
| **Neutrophil** | 2.08E+03 | 7.10E+04 | 1.27E+05 | 6.88E+04 | 4.75E+04 | 2.89E+04 | 7.26E+03 | 4.13E+04 | 5.62E+04 |
| **Ly6C^hi^ Mono/mac** | 1.64E+04 | 2.63E+04 | 8.59E+04 | 5.52E+04 | 8.97E+04 | 1.55E+04 | 2.30E+04 | 5.87E+04 | 1.17E+05 |
| **Ly6C^lo^ Mono/mac** | 2.94E+03 | 4.53E+03 | 2.27E+04 | 1.38E+04 | 2.77E+04 | 7.32E+03 | 5.57E+03 | 1.78E+04 | 3.98E+04 |
| **cDC1** | 2.40E+02 | 1.14E+03 | 5.82E+03 | 1.42E+03 | 2.26E+03 | 2.68E+02 | 8.51E+02 | 8.92E+03 | 4.16E+03 |
| **cDC2** | 9.08E+02 | 2.17E+03 | 1.03E+04 | 1.75E+03 | 5.03E+03 | 1.67E+03 | 2.12E+03 | 6.47E+03 | 8.44E+03 |

*Treg, regulatory T cell; DN, TCRβ^+^CD4^–^CD8^–^double negative T cell; MAIT, mucosal-associated invariant T cell; NKT, natural killer T cell; ILC, innate lymphoid cell; cNK, conventional NK cell; mono/mac, monocyte/macrophage; cDC, conventional dendritic cell.*

**Table S4. Mean absolute numbers of leukocyte subsets in the abdominal lymph nodes (~10-12 pooled).** Sample sizes (n) are as specified in main text Figures 1, 2, 4, 5 and 6.

|  | **Male** | | | | | | | **Female** | |
| --- | --- | --- | --- | --- | --- | --- | --- | --- | --- |
|  | **Balb.b** | **BALB/c** | **BALB/c Ly5.1** | **C57BL/6J** | **C57BL/6J Ly5.1** | **F1** | **DBA/2J** | **BALB/c** | **C57BL/6J** |
| **B cell** | 8.93E+05 | 3.96E+05 | 1.17E+06 | 4.76E+05 | 1.43E+06 | 2.69E+06 | 1.14E+06 | 2.22E+06 | 1.25E+06 |
| **CD4 T** | 5.06E+06 | 1.69E+06 | 1.67E+06 | 5.04E+05 | 1.27E+06 | 2.75E+06 | 2.22E+06 | 3.07E+06 | 1.03E+06 |
| **CD8αβ T** | 1.54E+06 | 7.18E+05 | 8.38E+05 | 6.09E+05 | 1.51E+06 | 2.47E+06 | 7.43E+05 | 1.31E+06 | 1.21E+06 |
| **Treg** | 6.86E+05 | 1.63E+05 | 2.64E+05 | 6.30E+04 | 2.24E+05 | 2.86E+05 | 1.80E+05 | 4.38E+05 | 1.61E+05 |
| **CD8αα T** | 1.17E+03 | 2.53E+02 | 9.97E+02 | 3.63E+03 | 8.56E+03 | 2.79E+03 | 5.83E+02 | 2.51E+03 | 5.07E+03 |
| **DN T** | 1.53E+04 | 5.10E+03 | 1.09E+04 | 1.25E+04 | 2.38E+04 | 1.52E+04 | 4.23E+03 | 1.41E+04 | 2.33E+04 |
| **MAIT** | 3.48E+03 | 6.58E+03 | 1.48E+03 | 6.85E+02 | 3.56E+03 | 1.27E+03 | 5.44E+02 | 4.93E+03 | 7.32E+03 |
| **NKT** | 1.61E+05 | 1.33E+04 | 6.45E+04 | 1.37E+03 | 8.32E+03 | 1.78E+04 | 7.89E+03 | 5.29E+04 | 1.10E+04 |
| **γδT** | 6.55E+04 | 7.52E+03 | 1.89E+04 | 2.28E+04 | 4.71E+04 | 2.87E+04 | 5.04E+04 | 3.46E+04 | 4.32E+04 |
| **ILC1** | 1.56E+03 | 4.02E+02 | 5.63E+02 | 1.86E+03 | 2.81E+03 | - | 1.12E+03 | 3.05E+02 | 2.35E+03 |
| **ILC2** | 3.91E+02 | 1.55E+02 | 2.32E+02 | 2.54E+02 | 4.90E+02 | - | 7.62E+02 | 2.19E+02 | 2.25E+02 |
| **ILC3** | 1.60E+02 | 2.94E+01 | 1.68E+01 | 1.06E+02 | 7.99E+00 | - | 7.48E+01 | 1.00E+02 | 6.84E+01 |
| **cNK** | 4.20E+04 | 1.03E+04 | 1.42E+04 | 1.09E+04 | 1.52E+04 | - | 3.14E+04 | 3.87E+04 | 1.80E+04 |
| **Eosinophil** | 1.84E+03 | 1.43E+02 | 4.01E+04 | 1.03E+03 | 8.37E+02 | 6.68E+01 | 2.65E+03 | 5.40E+02 | 4.13E+03 |
| **Neutrophil** | 6.71E+02 | 2.57E+02 | 3.15E+04 | 7.75E+02 | 8.69E+02 | 5.90E+01 | 2.92E+02 | 7.82E+02 | 1.18E+03 |
| **Ly6C^hi^ Mono/mac** | 3.87E+03 | 3.44E+02 | 2.45E+03 | 2.54E+03 | 6.80E+03 | 1.61E+02 | 1.51E+03 | 2.04E+03 | 3.41E+03 |
| **Ly6C^lo^ Mono/mac** | 1.68E+03 | 1.17E+02 | 1.14E+03 | 1.80E+03 | 2.52E+03 | 9.83E+01 | 2.34E+03 | 2.22E+03 | 1.29E+03 |
| **cDC1** | 2.73E+03 | 5.39E+02 | 5.32E+03 | 4.52E+03 | 7.26E+03 | 1.65E+02 | 2.33E+03 | 7.00E+03 | 4.11E+03 |
| **cDC2** | 6.46E+03 | 1.49E+02 | 1.38E+03 | 5.90E+03 | 1.06E+04 | 3.93E+02 | 3.18E+03 | 5.07E+03 | 6.87E+03 |

*Treg, regulatory T cell; DN, TCRβ^+^CD4^–^CD8^–^double negative T cell; MAIT, mucosal-associated invariant T cell; NKT, natural killer T cell; ILC, innate lymphoid cell; cNK, conventional NK cell; mono/mac, monocyte/macrophage; cDC, conventional dendritic cell. –, No data collected for the indicated group.*

**Table S5. Mean absolute numbers of leukocyte subsets in the spleen.** Sample sizes (n) are as specified in main text Figures 1, 2, 4, 5 and 6.

|  | **Male** | | | | | | | **Female** | |
| --- | --- | --- | --- | --- | --- | --- | --- | --- | --- |
|  | **Balb.b** | **BALB/c** | **BALB/c Ly5.1** | **C57BL/6J** | **C57BL/6J Ly5.1** | **F1** | **DBA/2J** | **BALB/c** | **C57BL/6J** |
| **B cell** | 9.38E+06 | 1.44E+07 | 2.44E+07 | 4.34E+07 | 4.22E+07 | 6.36E+07 | 3.06E+07 | 2.59E+07 | 3.54E+07 |
| **CD4 T** | 4.60E+06 | 6.23E+06 | 7.57E+06 | 7.99E+06 | 9.88E+06 | 1.25E+07 | 6.85E+06 | 7.55E+06 | 7.14E+06 |
| **CD8αβ T** | 2.31E+06 | 3.95E+06 | 3.58E+06 | 6.84E+06 | 8.94E+06 | 1.14E+07 | 2.53E+06 | 3.77E+06 | 6.62E+06 |
| **Treg** | 9.05E+05 | 8.20E+05 | 1.51E+06 | 1.38E+06 | 1.47E+06 | 1.60E+06 | 9.60E+05 | 1.28E+06 | 1.08E+06 |
| **CD8αα T** | 1.32E+04 | 6.98E+03 | 1.35E+04 | 9.72E+04 | 1.06E+05 | 4.25E+04 | 6.72E+03 | 2.49E+04 | 1.16E+05 |
| **DN T** | 1.10E+05 | 1.62E+05 | 2.63E+05 | 5.31E+05 | 5.42E+05 | 3.14E+05 | 1.57E+05 | 1.74E+05 | 5.26E+05 |
| **MAIT** | 1.16E+04 | 1.67E+04 | 2.22E+04 | 9.64E+03 | 2.59E+04 | 1.63E+04 | 7.56E+03 | 2.85E+04 | 5.32E+04 |
| **NKT** | 2.70E+05 | 6.19E+04 | 1.96E+05 | 1.73E+05 | 3.96E+05 | 4.84E+05 | 1.12E+05 | 3.77E+05 | 6.39E+05 |
| **γδT** | 2.29E+05 | 1.14E+05 | 2.75E+05 | 5.51E+05 | 5.33E+05 | 4.94E+05 | 4.00E+05 | 2.42E+05 | 5.81E+05 |
| **ILC1** | 3.63E+03 | 4.20E+03 | 1.25E+04 | 8.34E+04 | 2.53E+05 | 1.84E+04 | 1.39E+04 | 3.53E+03 | 3.41E+05 |
| **ILC2** | 1.84E+03 | 1.30E+03 | 2.59E+03 | 4.59E+03 | 3.66E+03 | 5.37E+03 | 5.41E+03 | 1.46E+03 | 2.30E+03 |
| **ILC3** | 2.36E+03 | 1.29E+03 | 2.04E+03 | 5.01E+03 | 1.69E+03 | 1.44E+03 | 1.54E+03 | 7.74E+02 | 1.83E+03 |
| **cNK** | 1.58E+06 | 1.60E+06 | 8.27E+05 | 2.19E+06 | 2.19E+06 | 4.17E+06 | 1.12E+06 | 1.81E+06 | 1.92E+06 |
| **Eosinophil** | 7.61E+04 | 6.97E+04 | 1.36E+05 | 2.87E+05 | 3.61E+05 | 4.62E+05 | 1.62E+05 | 1.15E+05 | 3.29E+05 |
| **Neutrophil** | 4.84E+05 | 6.52E+05 | 7.20E+05 | 6.58E+05 | 1.52E+06 | 2.16E+06 | 8.72E+05 | 9.86E+05 | 9.04E+05 |
| **Ly6C^hi^ Mono/mac** | 5.71E+05 | 2.89E+05 | 6.03E+05 | 6.92E+05 | 1.74E+06 | 1.35E+06 | 7.48E+05 | 3.63E+05 | 7.32E+05 |
| **Ly6C^lo^ Mono/mac** | 1.63E+05 | 8.63E+04 | 2.20E+05 | 3.26E+05 | 9.34E+05 | 7.18E+05 | 4.13E+05 | 1.76E+05 | 4.47E+05 |
| **cDC1** | 2.21E+04 | 3.79E+04 | 6.27E+04 | 8.91E+04 | 9.51E+04 | 7.59E+04 | 8.84E+04 | 9.89E+04 | 9.46E+04 |
| **cDC2** | 7.21E+04 | 4.70E+04 | 7.60E+04 | 6.09E+04 | 1.55E+05 | 2.07E+05 | 1.25E+05 | 1.68E+05 | 1.08E+05 |

*Treg, regulatory T cell; DN, TCRβ^+^CD4^–^CD8^–^double negative T cell; MAIT, mucosal-associated invariant T cell; NKT, natural killer T cell; ILC, innate lymphoid cell; cNK, conventional NK cell; mono/mac, monocyte/macrophage; cDC, conventional dendritic cell.*
